# Immune dysfunction prior to and during vaccination in multiple myeloma: a case study based on COVID-19

**DOI:** 10.1038/s41408-024-01089-5

**Published:** 2024-07-10

**Authors:** Esperanza Martín-Sánchez, Luis-Esteban Tamariz-Amador, Camila Guerrero, Anastasiia Zherniakova, Aintzane Zabaleta, Catarina Maia, Laura Blanco, Diego Alignani, Maria-Antonia Fortuño, Carlos Grande, Andrea Manubens, Jose-Maria Arguiñano, Clara Gomez, Ernesto Perez-Persona, Iñigo Olazabal, Itziar Oiartzabal, Carlos Panizo, Felipe Prosper, Jesus F. San-Miguel, Paula Rodriguez-Otero, Bruno Paiva, the Asociación Vasco-Navarra de Hematología y Hemoterapia (ASOVASNA) cooperative group, Jesus F. San Miguel

**Affiliations:** 1 Cancer Center Clinica Universidad de Navarra (CCUN), Centro de Investigacion Medica Aplicada (CIMA), Instituto de Investigacion Sanitaria de Navarra (IdiSNA), CIBER-ONC numbers CB16/12/00369 and CB16/12/00489, Pamplona, Spain; 2 Hospital Universitario de Navarra, IdiSNA, Pamplona, Spain; 3 Hospital Universitario de Galdakao, Galdakano, Spain; 4 Hospital Universitario de Araba—Txagorritxu, Vitoria-Gasteiz, Spain; 5 Hospital Universitario de Donostia, San Sebastian, Spain; 6 Hospital Universitario de Cruces, Bilbao, Spain

**Keywords:** Translational research, Tumour immunology, Myeloma

## Abstract

Infection is the leading cause of death in multiple myeloma (MM). However, the cellular composition associated with immune dysfunction is not defined. We analyzed immune profiles in the peripheral blood of patients with MM (*n* = 28) and B-cell chronic lymphoproliferative disorders (*n* = 53) vs. health care practitioners (*n* = 96), using multidimensional and computational flow cytometry. MM patients displayed altered distribution of most cell types (41/56, 73%), particularly within the B-cell (17/17) and T-cell (20/30) compartments. Using COVID-19 as a case study, we compared the immune response to vaccination based on 64,304 data points generated from the analysis of 1099 longitudinal samples. MM patients showed limited B-cell expansion linked to lower anti-RBD and anti-S antibody titers after the first two doses and booster. The percentages of B cells and CD4^+^ T cells in the blood, as well as the absolute counts of B cells and dendritic cells, predicted vaccine immunogenicity at different time points. In contrast with the humoral response, the percentage and antigen-dependent differentiation of SARS-CoV-2-specific CD8^+^ T cells was not altered in MM patients. Taken together, this study defined the cellular composition associated with immune dysfunction in MM and provided biomarkers such as the B-cell percentage and absolute count to individualize vaccination calendars.

## Introduction

Infection is the leading cause of morbidity and mortality in patients with multiple myeloma (MM) [1]. Studies in large cohorts showed a 7-fold increased risk of developing any infection compared to matched controls, both at diagnosis and during treatment, when patients are most immunosuppressed because of the cumulative effect of tumor and treatment-related factors [1, 2].

Approximately one out of five MM patients treated with older regimens were at risk of dying due to infection [1]. Such a risk is likely to increase with T-cell redirecting therapies [3, 4]. Recently, Lancman et al. reported that 41% of relapsed/refractory MM patients treated with BCMA-targeted bispecific antibodies experienced grade 3–5 infection, with an increasing cumulative probability over time, likely due to profound hypogammaglobulinemia [4]. Thus, the concern about infections in MM is probably greater than ever.

A recent example of the sequela of infections in MM was the coronavirus-19 disease (COVID-19). Compared to patients with solid tumors and individuals without cancer, the severe acute respiratory syndrome coronavirus 2 (SARS-CoV-2) caused more frequently serious clinical complications [5] and mortality was 50% higher in MM [6]. While COVID-19 vaccines ameliorated disease severity [7, 8] and reduced mortality rates [9], its immunogenicity is less potent and durable in MM [10]. Thus, it was suggested that patients were in need of more frequent boosters than the general population [11, 12].

It could be hypothesized that a more individualized vaccination based on the probability of immunogenicity at specific time points would be more effective than a prespecified calendar. Because the response to vaccination depends on host, tumor, and treatment-related factors, it may be presumed that immune profiling in blood could reflect its cumulative effect and be used as a minimally invasive routine test to predict vaccine efficacy at any time point. However, it remains unknown if the immune composition in blood truly captures the dysfunctional immunity of MM patients, and if biomarkers of adequate seroconversion can be readily identified through minimally invasive immune profiling. To address this, we performed a large-scale analysis that included two control groups in addition to MM patients and 64,304 data points generated from the analysis of 1099 longitudinal blood samples collected prior to and during COVID-19 vaccination.

## Results

### Patients’ characteristics

**Fig. 1:**
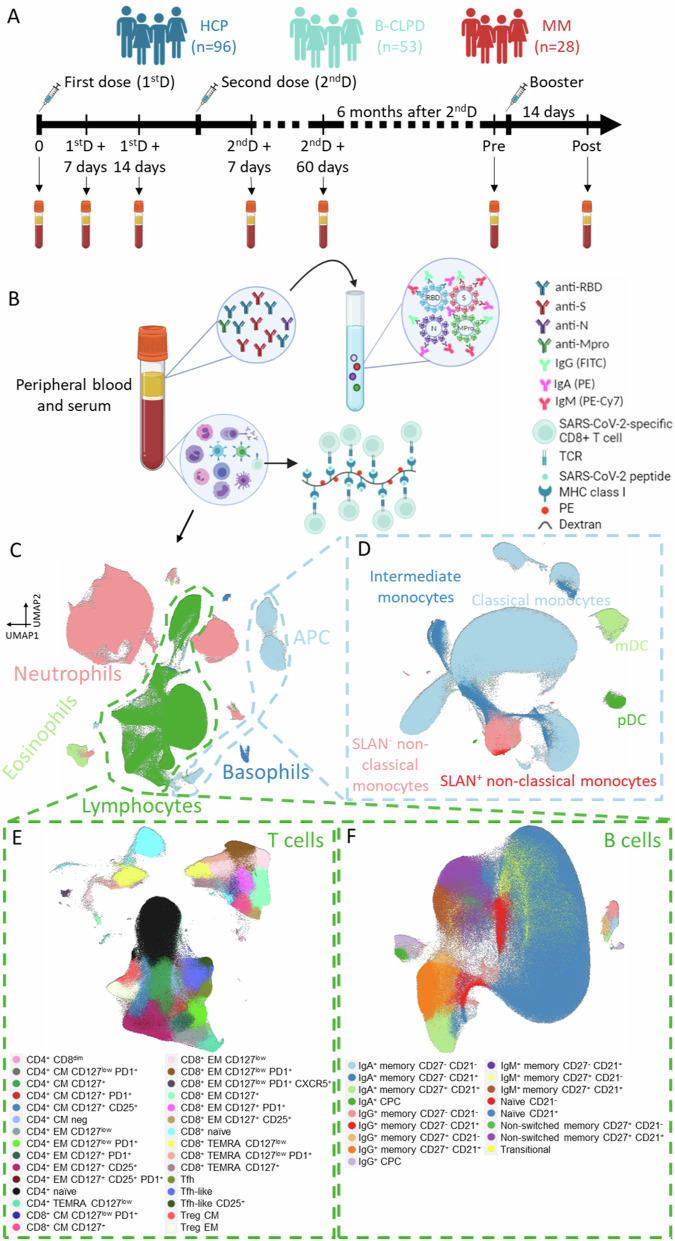
Study design. **A** Longitudinal analysis of peripheral blood and serum samples during COVID-19 vaccination in a total of 177 individuals: 28 patients with multiple myeloma (MM), 53 patients with a mature B-cell lymphoproliferative disorder (B-CLPD), and 96 health care practitioners (HCP) older than 50. Samples were drawn before vaccination, 7 and 14 days after the first dose, 7 and 60 days after the second dose, as well as before and 14 days after the booster. **B** In all samples, SARS-CoV-2-specific antibodies and CD8^+^ T-cells, as well as other non-virus-specific immune cells were analyzed. Antibodies were measured using a CE-IVD serological SARS-CoV-2 multiplex bead-based flow cytometry immunoassay, which allows the simultaneous and quantitative detection of specific IgM, IgG, and IgA antibodies to four SARS-CoV-2 antigens: (1) the receptor-binding domain (RBD) and (2) the stable trimer of the spike (S) glycoprotein; (3) the nucleocapsid (N) protein; and (4) the main viral protease (Mpro). Virus-specific CD8^+^ T-cells were detected by flow cytometry using a phycoerythrin (PE)-labeled dextran bound to MHC class I proteins presenting five viral peptides of the S-glycoprotein, membrane, ORF3a and N proteins. **C** Immune profiling was performed using multidimensional and computational flow cytometry that systematically identified a total of 56 immune cell types in peripheral blood, including basophils, eosinophils, neutrophils, antigen-presenting cells, and lymphocytes. **D** Antigen-presenting cells were sub-clustered into classical, intermediate, SLAN^−^ and SLAN^+^ non-classical monocytes, as well as plasmacytoid and myeloid dendritic cells (pDC and mDC, respectively). **E** Sub-clustering of T cells into 30 subsets related to antigen-dependent differentiation, along with activation and exhaustion phenotypes in helper and cytotoxic compartments. **F** Sub-clustering of B cells into 17 subsets related to antigen-dependent differentiation. CM central memory, CPC circulating plasma cells, EM effector memory, TEMRA effector memory T-cells re-expressing CD45RA, Tfh follicular helper T-cells, Treg regulatory T-cells.

This study included 28 patients with MM and two control groups: 53 cases with a B-cell chronic lymphoproliferative disorder (B-CLPD) and 96 health care practitioners (HCP) older than 50 years (Fig. 1A). The median age of MM patients was 61 years (range, 46-85) and sex distribution was 50:50 (Supplemental Table 1). Body mass index and the presence of comorbidities such as diabetes, hypertension, and autoimmunity were comparable across the three groups. By contrast, immunoparesis was more frequent in MM vs. B-CLPD (88% vs. 35%, *P* < 0.001). The median time since diagnosis was 4 years in both MM and B-CLPD. Most patients with MM were on treatment, while those with a B-CLPD were generally off-treatment (Supplemental Table 2); the depth of response at the time of vaccination was similar in both groups (Supplemental Table 1).

### Significantly altered immune composition in blood

Blood samples collected before vaccination (*n* = 177) were analyzed using multidimensional and computational flow cytometry to compare immune profiles in MM vs. B-CLPD and HCP (Fig. 1B). Accordingly, 56 immune subsets representative of the granulocytic, antigen-presenting, T and B cell compartments were systematically analyzed (Fig. 1C-F and supplemental Table 3).

**Fig. 2:**
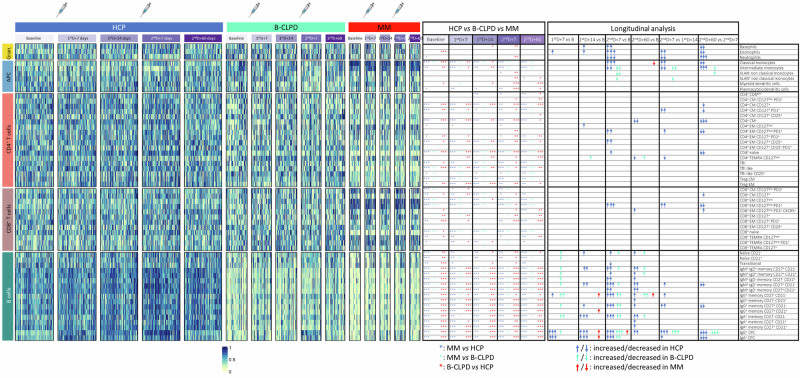
Immune composition in blood and response to vaccination. The 56 immune cell types were categorized into five main groups: granulocytes, antigen-presenting cells (APC), CD4 and CD8 T cells, as well as B cells. The percentile-defined frequency in the peripheral blood from 96 health care practitioners (HCP), 53 patients with a mature B-cell lymphoproliferative disorder (B-CLPD) and 28 patients with multiple myeloma (MM) is shown at baseline (B), 7 (1^st^D + 7) and 14 (1^st^D + 14) days after the first dose of the vaccine, and 7 (2^nd^D + 7) and 60 (2^nd^D + 60) days after the second dose. Each administration is indicated with a syringe. Blue, green, and red asterisks represent significant differences among groups, as indicated. A statistically significant increase or decrease in the frequency of immune cell types across time points in HCP (blue), B-CLPD patients (green), and MM patients (red) are indicated with up or down arrows that are respectively colored. A more detailed and graphical representation of such differences is shown in Supplemental Fig. 1. One symbol (asterisk or arrow), *P* < 0.05; two symbols, *P* < 0.01; three symbols, *P* < 0.001. CM central memory, CPC circulating plasma cells, EM effector memory, TEMRA terminally effector memory CD45RA^+^, Tfh follicular helper T-cells, Treg regulatory T-cells.

MM patients showed a significantly altered distribution of 41/56 (73%) immune subsets when compared to HCP (Fig. 2, Supplemental Fig. 1 and Supplemental Table 4). The B-cell compartment was the most altered, with reduced percentages of all 17 B-cell subsets, including naïve, antigen-experienced, and antibody-secreting cells of different isotypes. The T-cell compartment was the second most altered in MM (20 of 30 T-cell subsets). Nearly all CD4^+^ T cell types, including follicular-helper subsets, were reduced, whereas in the CD8^+^ compartment, there was a shift from lower percentages of naïve T cells towards an expansion of memory subsets, particularly those expressing CD127 (that is a marker for long-living memory T cells) and PD-1. The antigen-presenting cell compartment was the third most altered in MM due to reduced levels of plasmacytoid dendritic cells and an increased frequency of classical and intermediate monocytes. A reduced percentage of neutrophils was the only alteration observed within the granulocytic compartment. These results suggest that tumor and treatment-mediated B-cell depletion together with chronic antigen presentation to effector cells, which are classical hallmarks of immune dysfunction in MM, can be depicted through immune profiling of blood samples.

### The B-cell compartment does not expand in response to vaccination

We aimed at a longitudinal characterization of the immune response to the COVID-19 vaccine through the preplanned collection of blood and serum samples before vaccination, at days 7 and 14 after the first dose, at days 7 and 60 after the second dose, as well as before and 14 days after the booster (i.e., around 6 months since the second dose [Fig. 1A]). A total of 1099 samples were analyzed, and 64,304 data points were generated using multidimensional and computational flow cytometry (Fig. 1B–F).

The immune kinetics observed in HCP at days 7 and 14 after the first dose and days 7 and 60 after the second dose were used as a reference to interpret the immune response to vaccination in MM (and B-CLPD) patients (Fig. 2 and Supplemental Fig. 1). HCP showed an expansion of 11/17 B-cell subsets, which included naïve and memory B cells as well as circulating plasma cells, particularly of the IgG isotype. Only 4/30 T-cell subsets showed a significant expansion during vaccination: CD4 naïve and two effector memory subsets defined by differential expression of CD25, CD127, and PD-1, as well as CD8 effector memory T cells co-expressing CD127 and PD-1. Within the antigen presenting-cell compartment, there was an expansion of classical monocytes and a decrease in intermediate monocytes; whereas in the granulocytic compartment, basophils and eosinophils were expanded, with a progressive reduction of neutrophils until day 7 after the second dose.

In contrast to HCP and even to patients with B-CLPD who showed an expansion of the B-cell compartment similar to HCP (Fig. 2 and Supplemental Fig. 1), MM patients showed a limited response to vaccination. Only one of the 56 immune cell types (i.e., IgG^+^ circulating plasma cells) was significantly modulated at day 7 after the second dose, and none of the cellular kinetics identified in HCP was observed in MM. Thus, the altered immune profile of MM patients at baseline was followed by a limited response to vaccination.

### Link between a contracted B-cell compartment and limited antibody production

We next investigated if, in this cohort of MM patients there was a correlation between antibody production and the altered immune profile at baseline plus the limited response to vaccination. For this purpose, a total of 920 serum samples were analyzed at baseline, at day 7 after the first dose, and on days 7 and 60 after the second dose, using a CE-IVD serological SARS-CoV-2 multiplex bead-based flow cytometry immunoassay that simultaneously detected and quantified IgM, IgG, and IgA antibodies specific of four viral antigens (Fig. 1B).

**Fig. 3:**
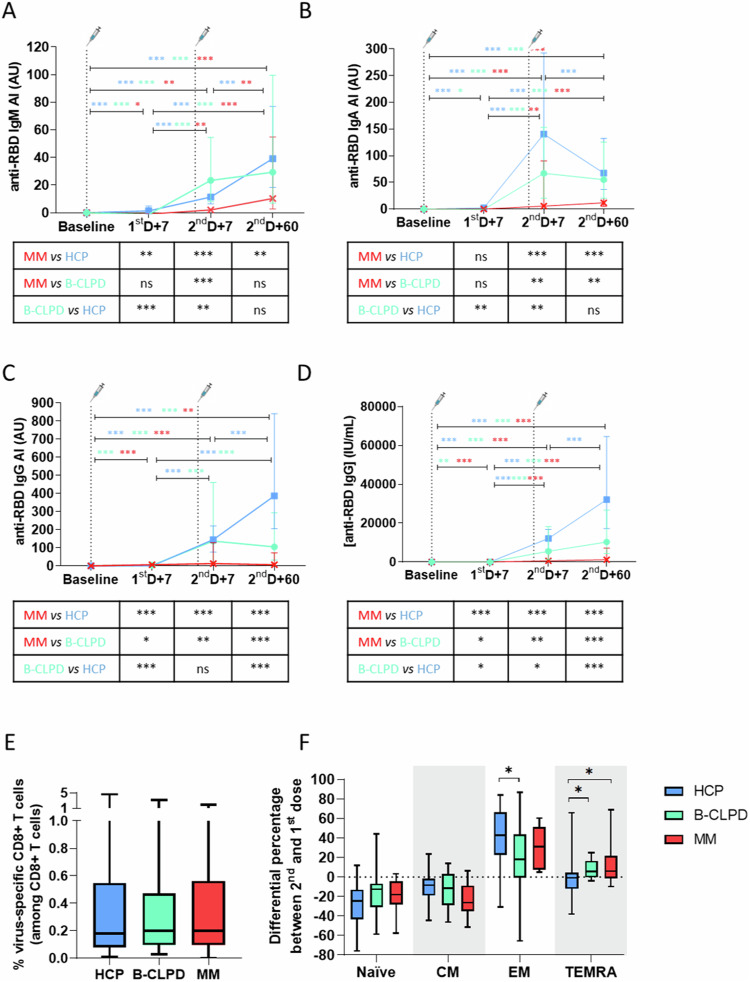
Antibody and cellular response to SARS-CoV-2 vaccination. Indexes of **A** IgM, **B** IgA, and **C** IgG antibodies, as well as **D** concentration of IgG against the receptor-binding domain (RBD) of the spike-glycoprotein were calculated at baseline, 7 days after the first dose (1^st^D + 7) as well as at days 7 (2^nd^D + 7) and 60 (2^nd^D + 60) after the second dose, in patients with multiple myeloma (MM, *n* = 28), a mature B-cell lymphoproliferative disorder (B-CLPD, *n* = 53), and health care practitioners (HCP, *n* = 96). Each administration is indicated with a syringe and a vertical line. Blue, green, and red asterisks indicate significant differences between the defined time points in HCP, B-CLPD patients, and MM patients, respectively. Black asterisks within tables show significant differences between groups of individuals at each time point. **E** SARS-CoV-2-specific CD8^+^ T-cells were identified one week after the second dose of the vaccine using a panel of five dextramers that bound to CD8^+^ T-cell receptor specific for the top five most immunodominant HLA-A*0201-restricted epitopes of the spike, membrane, ORF3a, and nucleocapsid viral proteins. This was performed in 70 individuals carrying the HLA-A2 allele: 34 HCP, 25 B-CLPD, and 11 MM patients. **F** Antigen-dependent differentiation of SARS-CoV-2-specific CD8^+^ T-cells was characterized 7 days after the first (1^st^D + 7) and second (2^nd^D + 7) dose of the vaccine using anti-CCR7 and anti-CD45RA antibodies to distinguish between naïve (CCR7^+^ CD45RA^+^), central memory (CM, CCR7^+^ CD45RA^-^), effector memory (EM, CCR7^−^ CD45RA^−^), and effector memory T-cells re-expressing CD45RA (TEMRA, CCR7^−^ CD45RA^+^). In all panels, one symbol, *P* < 0.05; two symbols, *P* < 0.01; three symbols, *P* < 0.001; ns non-significant, AI antibody index, AU arbitrary units.

MM patients systematically showed lower indexes of IgM, IgA, and IgG against the receptor-binding domain (RBD) of the spike viral antigen, which is the major target for virus-neutralizing antibodies [13]. At the time points of peak antibody production, anti-RBD IgM, IgA, and IgG levels were significantly lower in MM vs. B-CLPD patients and HCP (Fig. 3A–C). The concentration of anti-RBD IgG based on the WHO standards two months after the second dose was lower in MM vs*.* B-CLPD patients and HCP (1182 vs. 10,285 and 32,121 IU/mL, respectively, *P* < 0.001) (Fig. 3D). Similar results were observed for antibodies against the stable trimer of the S-glycoprotein (Supplemental Fig. 2). Thus, the inferior antibody production in MM was linked to the contraction at baseline and limited expansion throughout vaccination of the B-cell compartment.

### Preserved virus-specific T-cell response

The T-cell compartment was the second most altered at baseline and poorly responsive during vaccination in MM patients. Thus, we next investigated the magnitude of the virus-specific T-cell response in subjects carrying the HLA-A2 allele (11 MM, 25 B-CLPD, and 34 HCP). SARS-CoV-2-specific CD8^+^ T cells were quantified and characterized using multidimensional flow cytometry (Fig. 1B).

The median percentage of virus-specific CD8^+^ T cells detected in MM patients after the second dose was similar to that of B-CLPD and HCP (0.20%, 0.20%, and 0.18% among CD8^+^ T cells, respectively; *P* ≥ 0.71 for all comparisons) (Fig. 3E). Regarding the antigen-dependent differentiation of virus-specific CD8^+^ T cells from the first to the second vaccine dose, there was a significant expansion of effector memory T cells with a concomitant contraction of naïve and central memory T cells observed in all groups (Fig. 3F). Although the expansion of virus-specific CD8^+^ effector memory T cells in MM patients was not as pronounced as that observed in HCP, differences were not statistically significant (relative expansion of 31% *vs* 43%, respectively; *P* = 0.153). Similar results were observed with the booster (Supplemental Fig. 3). Taken together, these findings suggest that the fewer alterations in the T-cell compartment of MM patients correlated with an almost normal cellular response to vaccination.

### Cellular biomarkers predictive of vaccine immunogenicity

Because of the link between the magnitude of alterations in specific cell types and vaccine immunogenicity, we aimed at identifying minimally invasive biomarkers predictive of adequate antibody production. For this purpose and to facilitate the incorporation of such biomarkers in routine laboratory procedures, we regrouped the 56 immune subsets into the well-defined populations of basophils, eosinophils, neutrophils, classical, intermediate and non-classical monocytes, dendritic cells, CD4^+^ and CD8^+^ T cells, and B cells (Supplemental Table 5). Seroconversion was considered adequate if the anti-RBD IgG concentration after the second dose was equal to or greater than the median concentration observed in all patients (i.e., ≥ 4018 IU/mL).

**Fig. 4:**
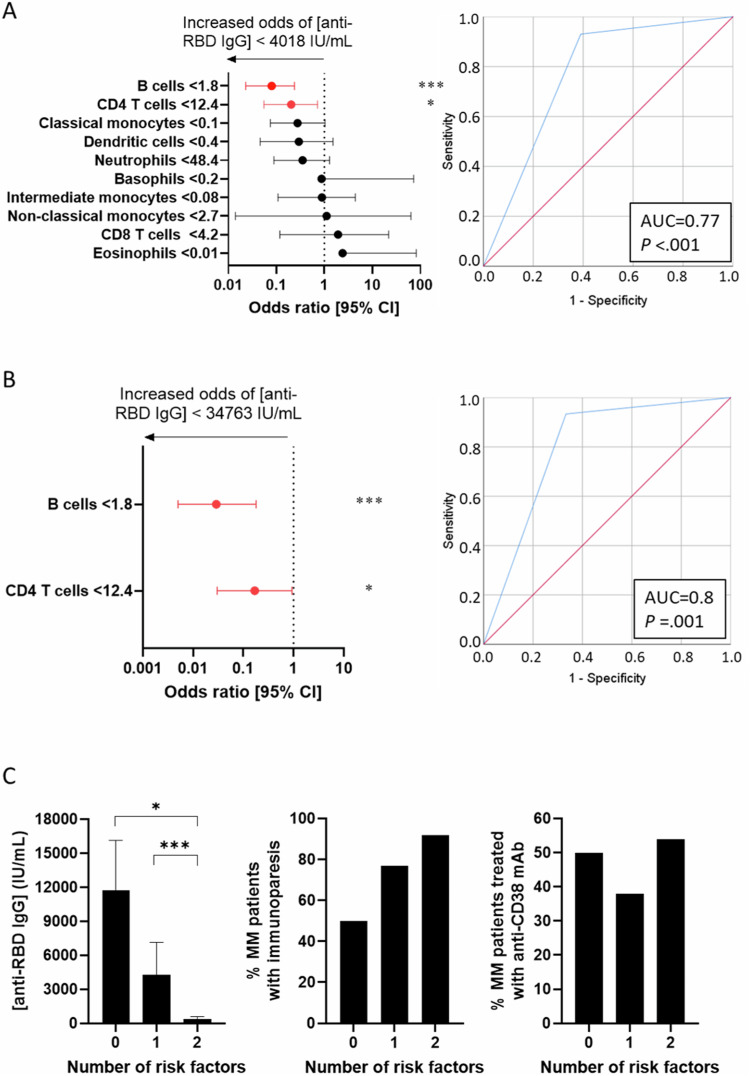
Cellular biomarkers predictive of vaccine immunogenicity. **A** Odds ratio multivariate analysis with 95% confidence intervals (CI) was included in the logistic regression model. After 10-fold cross-validation, the frequencies of B cells and CD4 T cells measured before vaccination significantly predicted inadequate seroconversion one week after the second dose, defined as below the median observed in patients at this time point (left panel). Area under the curve (AUC) of the prediction probabilities of the model in the training dataset (right panel). **B** Odds ratio multivariate analysis with 95% CI was included in the logistic regression for validation. Frequency of B cells and CD4 T cells measured before the booster administration significantly predicted inadequate seroconversion 14 days after the booster, defined as below the median of all patients at this time point (left panel). AUC of the prediction probabilities of the model in the validation dataset (right panel). **C** Association of the presence of none, one or two immune risk-factor predictive of inadequate seroconversion (left panel) with immunoparesis (middle panel) and treatment with anti-CD38 monoclonal antibodies (right panel).

Logistic regression with ten-fold cross-validation using optimal cutoffs (Supplemental Table 6) revealed that <1.8% B cells and <12.4% CD4^+^ T cells were significantly associated with inadequate seroconversion [mean area under the curve (AUC) = 0.77, *P* < 0.001] (Fig. 4A). Similar results were observed using absolute counts of B cells though not of CD4 T cells (Supplemental Table 6 and Supplemental Fig. 4). In addition, the number of dendritic cells was significantly associated with the magnitude of seroconversion.

To confirm these findings, we investigated if the percentage of the two cell types in the blood of MM patients before the booster was once again predictive of inadequate seroconversion. The immunogenicity of the booster was considered adequate if the anti-RBD IgG concentration was equal or greater than the median concentration observed in all patients (i.e., ≥34,763 IU/mL anti-RBD IgG).

Similar to the first vaccine doses, the booster had suboptimal efficacy in MM patients vs. HCP (Supplemental Fig. 5). MM patients displaying <1.8% B cells and <12.4% CD4^+^ T cells before the booster had a greater probability of achieving inadequate anti-RBD IgG concentration after (AUC = 0.8, *P* = 0.001) (Fig. 4B). Of note, the presence of none, one or two of these immune risk-factors predictive of lower seroconversion was associated with immunoparesis, but not with anti-CD38-based treatment (Fig. 4C). Thus, a simple flow cytometry test in blood assessing the percentages of B cells and CD4^+^ T cells could provide complementary information to predict vaccine immunogenicity at different time points.

## Discussion

The availability of newer immunotherapies has intensified research efforts to improve our understanding of the immune status of MM patients and to use that knowledge towards more individualized approaches with increased efficacy and decreased risk of adverse events such as severe infections. Here, we provide an atlas of the immune composition in the blood of MM patients and how it affects the efficacy of vaccination strategies such as for COVID-19.

Most studies of immune profiling in MM have analyzed bone marrow samples to characterize mechanisms associated with disease progression and treatment resistance [14–18]. By contrast, our understanding of the cellular architecture in blood is limited to fewer studies [19–21]. Here, we leveraged multidimensional and computational immune profiling to perform a comprehensive characterization of 56 subsets, most of which (73%) were found altered in MM compared to HCP older than 50 years. Thus, this study provides a measurement of the magnitude of MM immune dysfunction with regard to what can be expected from aging and related comorbidities in healthy adults without cancer. Because we analyzed a second control group of patients with a B-CLPD, our results also uncovered that MM was associated with more severe immune suppression. Thus, we suppose that the magnitude of immune dysfunction in the blood of MM patients may indeed reflect the cumulative effect of host, tumor, and treatment-related factors.

The consensus guidelines for infection prevention from the International Myeloma Working Group recommend an individualized treatment plan adapted by risk after comprehensive staging at diagnosis and relapse [22]. This includes taking the clinical history, examining physical health, and evaluating the functional status of older patients. Interestingly, a complete blood cell count is not recommended. Thus, the possibility that immune profiling in the blood may uncover immune suppressive states resulting from host, tumor, and/or treatment-related factors supports its potential role during staging. This should be investigated in future studies, which can leverage the median values and ranges of the immune subsets analyzed here (Supplemental Table 4). In this regard, we and others have shown recently how immune profiling in blood was an independent prognostic factor of severe SARS-CoV-2 infection in individuals with and without blood cancer [23–26]. This becomes particularly relevant with the advent of BCMA-targeting bispecific antibodies, which deplete (tumor and normal) plasma cells and are associated with an increased incidence of infections [27].

It is well-known that MM patients often show suboptimal response to vaccination and one recent example was the COVID-19 vaccine [10, 12]. While there is a growing understanding of the treatment-related factors associated with inferior immunogenicity [10, 28–30], little is known about the cellular dynamics during the immune response to vaccination. Here, we provided one of the most comprehensive analyses of the cellular dynamics during the immune response to vaccination, measured at five different time points before and after the first two doses. Albeit somehow expected, it was nonetheless stunning to see that most immune cells remained unresponsive in MM.

The B-cell compartment showed the greatest number of alterations, at baseline and during vaccination, which correlated with decreased antibody production. Of note, no severe infections or deaths from COVID-19 have yet been recorded in our cohort of MM patients, which is in accordance with other studies showing the positive impact of COVID-19 vaccination on morbidity and mortality rates [9, 31, 32]. Thus, while ours and the other studies provide evidence of how patients’ clinical status and B-cell numbers correlate with antibody titers, a better understanding of the causation behind protection against SARS-CoV-2 in the absence of adequate seroconversion may require more comprehensive and functional immune profiling and how it associates with the risk of severe infection.

An interesting observation of our study was that the T-cell compartment was less altered, which correlated with an adequate percentage of SARS-CoV-2 specific CD8^+^ T cells. These results build upon previous findings in patients with hematological cancer [33–36] that in the absence of adequate seroconversion, T-cell responses may protect vaccinated MM patients from severe infection. In consequence, the development of guidelines for the periodicity of boosters should probably consider the generation and persistence of virus-specific T cells in addition to antibody titers.

The International Myeloma Working Group recommends that the timing of vaccination should be individualized on the basis of the risks and benefits of immunization, including individual susceptibility to a specific infection, and the patient’s immune status [22]. However, the latter is inferred from the disease stage (i.e., patients with asymptomatic precursor conditions vs. active disease requiring therapy), previous treatment exposure (i.e., newly diagnosed vs. relapsed/refractory patients), and the type of therapy (e.g., high-dose melphalan with autologous transplant vs. an immune modulatory agent alone or with a proteasome inhibitor). There is only one reference to the recovery of CD4 cell count being a reasonable guide to immune recovery [22, 37–40].

Our results suggest that, as opposed to cell counts, only the percentage of CD4 T cells before vaccination significantly predicted the odds of an adequate seroconversion. Conversely, dendritic cell numbers but not percentages were predictive, whereas both the count and percentage of B cells were significantly associated with the magnitude of seroconversion. These findings are consistent with the recent observation that MM patients having an immune profile deficient in dendritic cells, NK cells, follicular-helper T cells, and B cells show suboptimal response to vaccination [41]. These immune biomarkers can be readily measured using routine flow cytometry, and such a minimally invasive test could help individualize current guidelines of empirical boosters every 6–12 months after the last shot or documented COVID-19 infection [12]. Such a strategy could potentially be expanded to other vaccine types according to the recent observations of immune determinants of influenza vaccine immunogenicity in pediatric hematopoietic cell transplant recipients [42]. We believe that such efforts are worthy to reduce the leading cause of morbidity and mortality in MM patients and continue improving clinical outcomes.

## Methods

### Study design

The baseline immune profile and response to COVID-19 vaccination were studied in a total of 177 individuals: 28 with MM, 53 with a mature B-CLPD, and 96 HCP older than 50 years. All subjects had no documented SARS-CoV-2 infection prior to vaccination. Peripheral blood and serum samples were preplanned to be collected before vaccination, at days 7 and 14 after the first dose, at days 7 and 60 after the second dose, as well as before and 14 days after the booster (around 6 months since the second dose [Fig. 1A]). The study was approved by the Ethics Committee of the University of Navarra (2021.006) and was conducted per the ethical principles of the Declaration of Helsinki. All subjects provided written informed consent before being enrolled in the study. Samples and data from patients and HCP were provided by the Biobank of the University of Navarra and were processed following standard operating procedures approved by the Ethical and Scientific Committees.

### Immune profiling

A total of 1099 peripheral blood samples were collected in EDTA-containing tubes and processed within 24 h following the EuroFlow lyse-wash-and-stain protocol (adjusted to 10^6^ nucleated cells) [43] (Fig. 1B). Three eight-color monoclonal antibody combinations (Supplemental Table 7) were designed to measure the antigen-dependent immune response of B and T cells, as well as to characterize the composition of granulocytes and antigen-presenting cells (APC) (Fig. 1C–F). Samples were measured in a FACSLyric flow cytometer (Beckton Dickinson Biosciences [BD], San Jose, CA) using FACSuite v1.3.0.6137 software (BD). Overall, 56 immune cell types were systematically evaluated in all subjects at every time point using computational flow cytometry (Supplemental Table 3 and Supplemental Fig. 6).

### Computational flow cytometry

Data were analyzed using the semi-automated workflow *FlowCT*, a workspace developed for the deconvolution of immunophenotypic data and objective reporting on large datasets [44]. Briefly, *FlowCT* performed internal data quality control, including correction of potential discrepancies in markers’ nomenclature, followed by data normalization, automated clustering, and dimensionality reduction. Clusters’ identities were manually annotated using the Infinicyt v2.0 software (Cytognos SL, Salamanca, Spain). Population abundances were subsequently exported to further calculate statistical differences among groups.

### Assessment of serological response

Antibody response to the COVID-19 vaccine was evaluated in a total of 920 serum samples, using a CE-IVD serological SARS-CoV-2 multiplex bead-based flow cytometry immunoassay (Immunostep SL, Salamanca, Spain) [45] to simultaneously detect and quantify in serum specific IgM, IgG and IgA antibodies to four viral antigens: the RBD and the stable trimer of the spike (S) glycoprotein, the nucleocapsid (N) protein, and the main virus protease (Mpro) (Fig. 1B). Based on the WHO First International Standard for Anti-SARS-CoV-2 Human Immunoglobulin (NIBSC Code 20/136) included in the assay, we also reported the concentration of anti-RBD or anti-S IgG in international units (IU/mL) or in binding antibody units (BAU/mL), respectively. The dilution range to obtain a linear regression model (*r* ≥ 0.94) was 1:1 to 1:16,384.

### Evaluation of cellular response

SARS-CoV-2-specific CD8^+^ T-cells were identified by flow cytometry using a Dextramer® panel of 5 epitopes of the S, N, membrane, and ORF3 viral proteins, which were presented by MHC class I molecules to T-cell receptor on virus-specific cells (Immudex, Virum, Denmark) (Fig. 1B). These epitopes were restricted to the HLA-A*0201 allele (the most prevalent and common in all human populations [46]), and were largely conserved in the predominant SARS-CoV-2 variants of concern in Spain during this study (Delta and Omicron). To discard potential false negative results due to non-matching HLA, all individuals were first subjected to HLA typing by flow cytometry using antibodies in supplemental Table 7, and virus-specific CD8^+^ T-cells were measured in subjects carrying the HLA-A2 allele (*n* = 70). Both frequency and phenotype of these cells were characterized one week after the first vaccine doses, as well as before and after the booster.

### Statistical analysis

The Kruskal–Wallis and Mann–Whitney tests were used to assess the statistical significance of differences in the relative distribution of granulocytic, antigen-presenting cell, CD4 and CD8 T-cell, as well as B-cell subsets, the frequency and phenotype of virus-specific CD8^+^ T cells, and antibody indexes and concentrations.

Logistic regression was performed to analyze the relationship between immune covariates in predicting adequate seroconversion to the second dose and the booster of the COVID-19 vaccine, respectively defined by the median concentrations found in all patients at each time point (i.e., ≥4018 and ≥34,763 IU/mL anti-RBD IgG). For this purpose, the 56 immune cell types were grouped into 10 main types (i.e., basophils, eosinophils, neutrophils, non-classical, intermediate and classical monocytes, dendritic cells, CD4 and CD8 T cells, as well as B cells). To maximize the area under the ROC curve, the J index was used to calculate cut-off values of relative and absolute counts before vaccination that were associated with adequate seroconversion one week after the second dose. After a 10-fold cross-validation performed by 10 random selections of 70% of individuals, a predictive model was generated based on significant immune variables associated with antibody response (*P* < 0.05). This model was further validated before the booster in 60 individuals. The frequency of significant immune cell types measured before the booster was used to stratify patients according to the same cut-off values identified above, and their potential in predicting adequate seroconversion after the booster was interrogated using logistic regression and ROC analyses. Statistical analyses were performed using SPSS (version 25.0.0, IBM, Armonk, NY), and R (version 4.0.0) software. *P*-values < 0.05 were considered significant.

### Supplementary information

Supplemental Material

## Data Availability

Data generated in this study that are unavailable within the article and its supplementary data files would be available upon a reasonable scientific request to the corresponding authors.
