# Supplementary material for: Immune dysfunction prior to and during vaccination in multiple myeloma: a case study based on COVID-19
Source: Blood Cancer J. 2024 Jul 10;14(1):111. doi: 10.1038/s41408-024-01089-5 (PMC11237013; doi:10.1038/s41408-024-01089-5)

**Supplemental Information**

[Supplemental Table 1 2](#_Toc169622962)

[Supplemental Table 2 4](#_Toc169622963)

[Supplemental Table 3 6](#_Toc169622964)

[Supplemental Table 4 10](#_Toc169622965)

[Supplemental Table 5 14](#_Toc169622966)

[Supplemental Table 6 15](#_Toc169622967)

[Supplemental Table 7 16](#_Toc169622968)

[Supplemental Figure 1 19](#_Toc169622969)

[Supplemental Figure 2 30](#_Toc169622970)

[Supplemental Figure 3 31](#_Toc169622971)

[Supplemental Figure 4 32](#_Toc169622972)

[Supplemental Figure 5 33](#_Toc169622973)

[Supplemental Figure 6 34](#_Toc169622974)

**SUPPLEMENTAL TABLES**

Supplemental Table 1. Epidemiological and clinical features of the subjects analyzed in this study.

| **Characteristics** | **MM patients** | **B-CLPD patients** | **HCP** |
| --- | --- | --- | --- |
| N | 28 | 53 | 96 |
| Age, median (range), years | 61 (46-85) | 54 (21-84) | 55 (50-70) |
| Female, No. (%) | 14 (50%) | 22 (42%) | 88 (92%) |
| **Comorbidities, No. (%)** |  |  |  |
| Any | 13 (46%) | 19 (36%) | 31 (32%) |
| Body Mass Index > 30 | 4 (14%) | 9 (17%) | 9 (9%) |
| Diabetes | 4 (14%) | 2 (4%) | 0 (0%) |
| Arterial hypertension | 7 (25%) | 4 (8%) | 8 (8%) |
| Autoimmune disease | 2 (7%) | 1 (2%) | 6 (6%) |
| **Type of vaccine, No. (%)** |  |  |  |
| BNT162b2 | 14 (50%) | 36 (68%) | 28 (29%) |
| mRNA-1273 | 14 (50%) | 17 (32%) | 68 (71%) |
| Time since diagnosis, median (range), years | 4.0 (1-10) | 4.2 (2-28) | - |
| **Treatment situation, No. (%)** |  |  |  |
| No treatment | 2 (7%) | 14 (26%) | - |
| On treatment | 15 (54%) | 4 (8%) | - |
| Off treatment | 11 (39%) | 35 (66%) | - |
| **Type of treatment, No. (%)** |  |  |  |
| anti-CD38 mAb | 13 (50%) | 0 (0%) | - |
| anti-CD20 mAb | 0 (0%) | 33 (85%) | - |
| Autologous transplant | 15 (58%) | 5 (13%) | - |
| Immunomodulatory agent | 21 (81%) | 0 (0%) | - |
| Previous lines of treatment, median (range) | 2 (1-5) | 1 (1-4) | - |
| Relapsed/refractory to first line, No. (%) | 16 (62%) | 6 (15%) | - |
| Complete response, No. (%) | 20 (77%) | 31 (79%) | - |
| Immunoparesis, No. (%) | 23 (88%) | 8 (35%) | - |

MM, multiple myeloma; B-CLPD, mature B-cell lymphoproliferative disorder; HCP; health care practitioners; mAb, monoclonal antibody.

Supplemental Table 2. Current and previous lines of treatment at the time of vaccination and seroconversion status after vaccination. The number of months elapsing between autologous stem cell transplantation (ASCT) and the time of vaccination is shown in parenthesis.

| **Patient ID** | **Previous lines** | | | **Current line** | **Adequate seroconv.** |
| --- | --- | --- | --- | --- | --- |
|  | **1st** | **2nd** | **3rd** |  |  |
| MM107 | Vd/ASCT (101) | VRd | - | DRd | No |
| MM110 | VRd/ASCT (53) | Dara-Vd | - | DPd | No |
| MM112 | VBCMP/VBAD | VCd | KRd/ASCT (23) | DRd | No |
| MM117 | VCd | - | - | KRd | No |
| MM118 | VTd | Rd | VCd | Rd | No |
| MM131 |  | - | - | Dara-VRd/ASCT/R (13) | Yes |
| MM132 |  | - | - | Dara-VRd | No |
| MM140 | VBCMP/VBAD | VCd | KRd | DRd | No |
| MM171 |  | - | - | VRd/ASCT/R (21) | Yes |
| MM180 | VBCMP/VBAD/ ASCT (26) | - | - | VCd+R | No |
| MM187 |  | - | - | VCd/ASCT/R (17) | Yes |
| MM188 | Dara-VRd/ASCT (9) | KCD | - | VRD-PACE | No |
| MM190 |  | - | - | VRd | No |
| MM191 |  | - | - | VCd/ASCT (4) | Yes |
| MM201 |  | - | - | VRd | No |
| B-CLPD123 | R-CHOP | R-Bendamustine | Obinutuzumab-Bendamustine | Mosunetuzumab | No |
| B-CLPD126 | R-CHOP/HDAC/ ASCT (4) |  | - | Methothrexate + Ibrutinib | No |
| B-CLPD143 |  | - | - | R-Bendamustine | No |
| B-CLPD181 |  | - | - | R-Da-EPOCH | No |

ASCT, autologous stem cell transplant; VD, Bortezomib + Dexamethasone; VRd, Bortezomib + Lenalidomide + Dexamethasone; DRd, Daratumumab + Lenalidomide + Dexamethasone; DPd, Daratumumab + Pomalidomide + Dexamethasone; Rd, Lenalidomide + Dexamethasone; VCd, Bortezomib + Cyclophosphamide + Dexamethasone; VBCMP/VBAD, Vincristine + Carmustine + Cyclophosphamide + Melphalan + Prednisone / Vincristine + Camustine + Doxorubicin + Dexamethasone; Dara-Vd, Daratumumab + Bortezomib + Dexamethasone; Dara-VRd, Daratumumab + Bortezomib + Lenalidomide + Dexamethasone; VTd, Bortezomib + Thalidomide + Dexamethasone; KRd, Carfilzomib + Lenalidomide + Dexamethasone; KCd, Carfilzomib + Cyclophosphamide + Dexamethasone; VRd-PACE, Bortezomib + Lenalidomide + Dexamethasone + Cyclophosphamide + Cisplatin + Doxorubicin + Etoposide; R-CHOP, Rituximab + Cyclophosphamide + Doxorubicin + Vincristine + Prednisone; R-Bendamustine, Rituximab-Bendamustine; R-Da-EPOCH, Rituximab + Etoposide + Prednisone + Vincristine + Doxorubicin; R-CHOP/HDAC, Rituximab + Cyclophosphamide + Doxorubicin + Vincristine + Prednisone / High dose cytarabine

Supplemental Table 3. Immunophenotypic profile of the 56 immune cell types identified by automated clustering using *FlowCT*.

| **Immune subset** | **Immune cell type** | **Immunophenotype** |
| --- | --- | --- |
| **Granulocytes** | Basophils | SSC^lo^ FSC^hi^ CD45^lo^ CD33^+^ CD16^-^ HLA-DR^-^ CD123^+^ |
|  | Eosinophils | SSC^hi^ FSC^hi^ CD45^hi^ CD33^+^ CD16^-^ HLA-DR^-^ CD123^-^ |
|  | Neutrophils | SSC^hi^ FSC^hi^ CD45^+^ CD33^het^ CD16^hi^ HLA-DR^-^ CD123^-^ |
| **APC** | Classical monocytes | SSC^lo^ FSC^lo^ CD45^hi^ CD33^+^ CD16^-^ CD14^+^ CD36^+^ HLA-DR^+^ CD123^-^ SLAN^-^ |
|  | Intermediate monocytes | SSC^lo^ FSC^lo^ CD45^hi^ CD33^+^ CD16^+^ CD14^+^ CD36^+^ HLA-DR^hi^ CD123^-^ SLAN^-^ |
|  | SLAN^-^ non-classical monocytes | SSC^lo^ FSC^lo^ CD45^hi^ CD33^+^ CD16^+^ CD14^-^ CD36^+^ HLA-DR^hi^ CD123^-^ SLAN^-^ |
|  | SLAN^+^ non-classical monocytes | SSC^lo^ FSC^lo^ CD45^hi^ CD33^+^ CD16^+^ CD14^-^ CD36^+^ HLA-DR^hi^ CD123^-^ SLAN^+^ |
|  | Myeloid dendritic cells (mDC) | SSC^lo^ FSC^lo^ CD45^hi^ CD33^+^ CD16^-^ CD14^-^ CD36^lo^ HLA-DR^hi^ CD123^-^ SLAN^-^ |
|  | Plasmacytoid dendritic cells (pDC) | SSC^lo^ FSC^lo^ CD45^hi^ CD33^lo^ CD16^-^ CD14^-^ CD36^lo^ HLA-DR^+^ CD123^+^ SLAN^-^ |
| **CD4^+^ T-cells** | CD4^+^ CD8^dim^ | CD4^+^ CD8^dim^ CCR7^+^ CD45RA^+^ CD127^+^ CD25^het^ PD1^-^ CXCR5^-^ |
|  | CD4^+^ naïve | CD4^+^ CD8^-^ CCR7^+^ CD45RA^+^ CD127^+^ CD25^het^ PD1^-^ CXCR5^-^ |
|  | CD4^+^ CM^-^ | CD4^+^ CD8^-^ CCR7^+^ CD45RA^-^ CD127^-^ CD25^-^ PD1^-^ CXCR5^-^ |
|  | CD4^+^ CM CD127^+^ | CD4^+^ CD8^-^ CCR7^+^ CD45RA^-^ CD127^+^ CD25^-^ PD^1+^ CXCR5^-^ |
|  | CD4^+^ CM CD127^+^ CD25^+^ | CD4^+^ CD8^-^ CCR7^+^ CD45RA^-^ CD127^+^ CD25^+^ PD1^+^ CXCR5^-^ |
|  | CD4^+^ CM CD127^lo^ PD1^+^ | CD4^+^ CD8^-^ CCR7^+^ CD45RA^-^ CD127^lo^ CD25^-^ PD1^+^ CXCR5^-^ |
|  | CD4^+^ CM CD127^+^ PD1^+^ | CD4^+^ CD8^-^ CCR7^+^ CD45RA^-^ CD127^+^ CD25^-^ PD1^+^ CXCR5^-^ |
|  | CD4^+^ EM CD127^lo^ | CD4^+^ CD8^-^ CCR7^-^ CD45RA^+^ CD127^lo^ CD25^-^ PD1^-^ CXCR5^-^ |
|  | CD4^+^ EM CD127^+^ CD25^+^ | CD4^+^ CD8^-^ CCR7^-^ CD45RA^-^ CD127^+^ CD25^+^ PD1^-^ CXCR5^-^ |
|  | CD4^+^ EM CD127^lo^ PD1^+^ | CD4^+^ CD8^-^ CCR7^-^ CD45RA^-^ CD127^lo^ CD25^-^ PD1^+^ CXCR5^-^ |
|  | CD4^+^ EM CD127^+^ PD1^+^ | CD4^+^ CD8^-^ CCR7^-^ CD45RA^-^ CD127^+^ CD25^-^ PD1^+^ CXCR5^-^ |
|  | CD4^+^ EM CD127^+^ CD25^+^ PD1^+^ | CD4^+^ CD8^-^ CCR7^-^ CD45RA^-^ CD127^+^ CD25^+^ PD1^+^ CXCR5^-^ |
|  | CD4^+^ TEMRA CD127^lo^ | CD4^+^ CD8^-^ CCR7^-^ CD45RA^+^ CD127^lo^ CD25- PD1^-^ CXCR5^-^ |
|  | CM Treg | CD4^+^ CD8^-^ CCR7^+^ CD45RA^-^ CD127^lo^ CD25^+^ PD1^-^ CXCR5^-^ |
|  | EM Treg | CD4^+^ CD8^-^ CCR7^-^ CD45RA^-^ CD127^lo^ CD25^+^ PD1^-^ CXCR5^-^ |
|  | Tfh | CD4^+^ CD8^-^ CCR7^+^ CD45RA^-^ CD127^+^ CD25^-^ PD1^+^ CXCR5^+^ |
|  | Tfh-like | CD4^+^ CD8^-^ CCR7^+^ CD45RA^-^ CD127^+^ CD25^-^ PD1^-^ CXCR5^+^ |
|  | Tfh-like CD25^+^ | CD4^+^ CD8^-^ CCR7^+^ CD45RA^-^ CD127^+^ CD25^+^ PD1^-^ CXCR5^+^ |
| **CD8^+^ T-cells** | CD8^+^ naïve | CD4^-^ CD8^+^ CCR7^+^ CD45RA^+^ CD127^+^ CD25^-^ PD1^-^ CXCR5^-^ |
|  | CD8^+^ CM CD127^lo^ PD1^+^ | CD4^-^ CD8^+^ CCR7^+^ CD45RA^-^ CD127^lo^ CD25^-^ PD1^+^ CXCR5^-^ |
|  | CD8^+^ CM CD127^+^ | CD4^-^ CD8^+^ CCR7^+^ CD45RA^-^ CD127^+^ CD25^-^ PD1^-^ CXCR5^-^ |
|  | CD8^+^ EM CD127^lo^ | CD4^-^ CD8^+^ CCR7^-^ CD45RA^-^ CD127^lo^ CD25^-^ PD1^-^ CXCR5^-^ |
|  | CD8^+^ EM CD127^+^ | CD4^-^ CD8^+^ CCR7^-^ CD45RA^-^ CD127^+^ CD25^-^ PD1^-^ CXCR5^-^ |
|  | CD8^+^ EM CD127^lo^ PD1^+^ | CD4^-^ CD8^+^ CCR7^-^ CD45RA^-^ CD127^lo^ CD25^-^ PD1^+^ CXCR5^-^ |
|  | CD8^+^ EM CD127^lo^ PD1^+^ CXCR5^+^ | CD4^-^ CD8^+^ CCR7^-^ CD45RA^-^ CD127^lo^ CD25^-^ PD1^+^ CXCR5^+^ |
|  | CD8^+^ EM CD127^+^ PD1^+^ | CD4^-^ CD8^+^ CCR7^-^ CD45RA^-^ CD127^+^ CD25^-^ PD1^+^ CXCR5^-^ |
|  | CD8^+^ EM CD127^+^ CD25^+^ | CD4^-^ CD8^+^ CCR7^-^ CD45RA^-^ CD127^+^ CD25^+^ PD1^+^ CXCR5^-^ |
|  | CD8^+^ TEMRA CD127^lo^ | CD4^-^ CD8^+^ CCR7^-^ CD45RA^+^ CD127^lo^ CD25^-^ PD1^-^ CXCR5^-^ |
|  | CD8^+^ TEMRA CD127^lo^ PD1^+^ | CD4^-^ CD8^+^ CCR7^-^ CD45RA^+^ CD127^lo^ CD25^-^ PD1^+^ CXCR5^-^ |
|  | CD8^+^ TEMRA CD127^+^ | CD4^-^ CD8^+^ CCR7^-^ CD45RA^+^ CD127^+^ CD25^-^ PD1^-^ CXCR5^-^ |
| **B-cells** | Naïve CD21^-^ | CD27^-^ CD38^-^ CD21^-^ IgD^+^ IgM^+^ IgA^-^ IgG^-^ IgE^-^ |
|  | Naïve CD21^+^ | CD27^-^ CD38^-^ CD21^+^ IgD^+^ IgM^+^ IgA^-^ IgG^-^ IgE^-^ |
|  | Transitional | CD27^-^ CD38^+^ CD21^+^ IgD^-^ IgM^lo^ IgA^-^ IgG^-^ IgE^-^ |
|  | IgM^+^ IgD^+^ memory CD27^+^ CD21^-^ | CD27^+^ CD38^-^ CD21^-^ IgD^+^ IgM^+^ IgA^-^ IgG^-^ IgE^-^ |
|  | IgM^+^ IgD^+^ memory CD27^+^ CD21^+^ | CD27^+^ CD38^-^ CD21^-^ IgD^+^ IgM^+^ IgA^-^ IgG^-^ IgE^-^ |
|  | IgM^+^ IgD^-^ memory CD27^-^ CD21^+^ | CD27^-^ CD38^-^ CD21^+^ IgD^-^ IgM^+^ IgA^-^ IgG^-^ IgE^-^ |
|  | IgM^+^ IgD^-^ memory CD27^+^ CD21^-^ | CD27^+^ CD38^-^ CD21^-^ IgD^-^ IgM^+^ IgA^-^ IgG^-^ IgE^-^ |
|  | IgM^+^ IgD^-^ memory CD27^+^ CD21^+^ | CD27^+^ CD38^-^ CD21^+^ IgD^-^ IgM^+^ IgA^-^ IgG^-^ IgE^-^ |
|  | IgG^+^ memory CD27^-^ CD21^-^ | CD27^-^ CD38^-^ CD21^-^ IgD^-^ IgM^-^ IgA^-^ IgG^+^ IgE^-^ |
|  | IgG^+^ memory CD27^-^ CD21^+^ | CD27^-^ CD38^-^ CD21^+^ IgD^-^ IgM^-^ IgA^-^ IgG^+^ IgE^-^ |
|  | IgG^+^ memory CD27^+^ CD21^-^ | CD27^+^ CD38^-^ CD21^-^ IgD^-^ IgM^-^ IgA^-^ IgG^+^ IgE^-^ |
|  | IgG^+^ memory CD27^+^ CD21^+^ | CD27^+^ CD38^-^ CD21^+^ IgD^-^ IgM^-^ IgA^-^ IgG^+^ IgE^-^ |
|  | IgA^+^ memory CD27^-^ CD21^-^ | CD27^-^ CD38^-^ CD21^-^ IgD- IgM^-^ IgA^+^ IgG^-^ IgE^-^ |
|  | IgA^+^ memory CD27^-^ CD21^+^ | CD27^-^ CD38^-^ CD21^+^ IgD^-^ IgM^-^ IgA^+^ IgG^-^ IgE^-^ |
|  | IgA^+^ memory CD27^+^ CD21^+^ | CD27^+^ CD38^-^ CD21^+^ IgD^-^ IgM^-^ IgA^+^ IgG^-^ IgE^-^ |
|  | IgG^+^ circulating PC | CD27^hi^ CD38^hi^ CD21^-^ IgD^-^ IgM^-^ IgA^-^ IgG^+^ IgE^-^ |
|  | IgA^+^ circulating PC | CD27^hi^ CD38^hi^ CD21^-^ IgD^-^ IgM^-^ IgA^+^ IgG^-^ IgE^-^ |

APC, antigen-presenting cells; CM, central memory; EM, effector memory; TEMRA, effector memory T-cell re-expressing CD45RA; Tfh, follicular helper T cell, Treg, regulatory T cell; Ig, immunoglobulin; PC, plasma cells.

Supplemental Table 4. Baseline distribution of the 56 immune cell types in peripheral blood of patients with multiple myeloma (MM, n=28), patients with a B-cell lymphoproliferative disorder (B-CLPD, n=53), and health care practitioners (HCP, n=96). Numbers indicate the median percentage along with the range.

| **Immune subset** | **Immune cell type** | **MM** | **B-CLPD** | **HCP** |
| --- | --- | --- | --- | --- |
| **Granulocytes** | Basophils | 0.8 (0.0-4.2) | 0.8 (0.1-2.6) | 0.8 (0.0-2.4) |
|  | Eosinophils | 1.5 (0.2-10.3) | 1.8 (0.0-8.4) | 1.0 (0.0-4.0) |
|  | Neutrophils | 47.6 (20.8-73.7) | 54.9 (3.9-76.0) | 55.0 (32.8-83.0) |
| **APC** | Classical monocytes | 11.7 (5-41.4) | 6.9 (0.3-21.0) | 5.7 (0.0-10.6) |
|  | Intermediate monocytes | 0.4 (0.0-4.5) | 0.4 (0.0-5.7) | 0.2 (0.0-11.9) |
|  | SLAN^-^ non-classical monocytes | 0.3 (0.0-1.6) | 0.5 (0.0-2.2) | 0.5 (0.1-1.4) |
|  | SLAN^+^ non-classical monocytes | 0.0 (0.0-0.1) | 0.0 (0.0-0.4) | 0.0 (0.0-0.8) |
|  | Myeloid dendritic cells (mDC) | 0.3 (0.1-1.0) | 0.2 (0.0-1.8) | 0.3 (0.0-0.6) |
|  | Plasmacytoid dendritic cells (pDC) | 0.1 (0.0-0.4) | 0.1 (0.0-0.9) | 0.2 (0.0-0.4) |
| **CD4^+^ T-cells** | CD4^+^ CD8^dim^ | 0.0 (0.0-0.3) | 0.0 (0.0-0.3) | 0.0 (0.0-0.8) |
|  | CD4^+^ naïve | 0.3 (0.0-8.3) | 2.3 (0.0-23.3) | 5.7 (1.0-12.6) |
|  | CD4^+^ CM^-^ | 0.2 (0.0-0.7) | 0.2 (0.0-1.4) | 0.3 (0.0-0.9) |
|  | CD4^+^ CM CD127^+^ | 0.9 (0.1-9.0) | 0.8 (0.0-4.9) | 2.1 (0.5-5.7) |
|  | CD4^+^ CM CD127^+^ CD25^+^ | 0.4 (0.0-2.3) | 0.8 (0.0-3.7) | 0.7 (0.0-3.4) |
|  | CD4^+^ CM CD127^lo^ PD1^+^ | 0.1 (0.0-0.5) | 0.0 (0.0-5.2) | 0.1 (0.0-1.0) |
|  | CD4^+^ CM CD127^+^ PD1^+^ | 0.1 (0.0-0.7) | 0.2 (0.0-2.9) | 0.3 (0.0-1.3) |
|  | CD4^+^ EM CD127^lo^ | 0.7 (0.1-4.2) | 0.3 (0.0-12.4) | 0.5 (0.0-3.4) |
|  | CD4^+^ EM CD127^+^ CD25^+^ | 1.2 (0.1-3.3) | 1.7 (0.2-9.8) | 1.9 (0.7-4.8) |
|  | CD4^+^ EM CD127^lo^ PD1^+^ | 0.5 (0.1-3.7) | 0.3 (0.0-2.9) | 0.4 (0.0-1.8) |
|  | CD4^+^ EM CD127^+^ PD1^+^ | 0.2 (0.0-1.3) | 0.2 (0.0-1.7) | 0.4 (0.0-2.5) |
|  | CD4^+^ EM CD127^+^ CD25^+^ PD1^+^ | 0.1 (0.0-0.4) | 0.1 (0.0-0.8) | 0.1 (0.0-1.1) |
|  | CD4^+^ TEMRA CD127^lo^ | 0.1 (0.0-4.6) | 0.1 (0.0-3.8) | 0.1 (0.0-3.8) |
|  | CM Treg | 0.2 (0.0-0.5) | 0.3 (0.0-1.0) | 0.4 (0.0-1.2) |
|  | EM Treg | 0.4 (0.1-1.1) | 0.4 (0.0.-1.5) | 0.6 (0.0-1.5) |
|  | Tfh | 0.1 (0.0-0.7) | 0.2 (0.0-1.4) | 0.3 (0.0-1.0) |
|  | Tfh-like | 0.3 (0.0-1.8) | 0.6 (0.0-3.6) | 1.0 (0.2-3.2) |
|  | Tfh-like CD25^+^ | 0.1 (0.0-0.6) | 0.3 (0.0-1.1) | 0.3 (0.0-1.2) |
| **CD8^+^ T-cells** | CD8^+^ naïve | 0.4 (0.0-3.0) | 1.3 (0.1-9.8) | 1.7 (0.5-6.5) |
|  | CD8^+^ CM CD127^lo^ PD1^+^ | 0.2 (0.0-0.8) | 0.1 (0.0-1.2) | 0.1 (0.0-0.9) |
|  | CD8^+^ CM CD127^+^ | 0.2 (0.0-1.2) | 0.3 (0.0-1.3) | 0.4 (0.1-1.7) |
|  | CD8^+^ EM CD127^lo^ | 3.6 (0.1-13.2) | 0.7 (0.1-6.8) | 0.5 (0.0-6.9) |
|  | CD8^+^ EM CD127^lo^ PD1^+^ | 1.4 (0.1-9.5) | 0.3 (0.1-2.2) | 0.4 (0.0-1.9) |
|  | CD8^+^ EM CD127^lo^ PD1^+^ CXCR5^+^ | 0.1 (0.0-0.3) | 0.1 (0.0-0.3) | 0.1 (0.0-0.4) |
|  | CD8^+^ EM CD127^+^ | 1.4 (0.0-7.3) | 0.7 (0.1-3.7) | 1.0 (0.2-3.7) |
|  | CD8^+^ EM CD127^+^ PD1^+^ | 0.3 (0.0-2.7) | 0.2 (0.0-2.3) | 0.5 (0.0-1.9) |
|  | CD8^+^ EM CD127^+^ CD25^+^ | 0.1 (0.0-0.5) | 0.2 (0.0-1.2) | 0.1 (0.0-2.0) |
|  | CD8^+^ TEMRA CD127^lo^ | 1.1 (0.0-12.4) | 0.7 (0.0-8.1) | 0.4 (0.0-4.5) |
|  | CD8^+^ TEMRA CD127^lo^ PD1^+^ | 0.2 (0.0-3.9) | 0.1 (0.0-1.7) | 0.1 (0.0-2.6) |
|  | CD8^+^ TEMRA CD127^+^ | 0.2 (0.0-2.9) | 0.3 (0.1-1.8) | 0.2 (0.0-1.8) |
| **B-cells** | Naïve CD21^-^ | 0.0 (0.0-0.2) | 0.0 (0.0-0.2) | 0.0 (0.0-1.0) |
|  | Naïve CD21^+^ | 0.1 (0.0-4.7) | 1.2 (0.0-6.0) | 1.8 (0.0-16.6) |
|  | Transitional | 0.0 (0.0-0.7) | 0.1 (0.0-0.5) | 0.2 (0.0-2.1) |
|  | IgM^+^ IgD^+^ memory CD27^+^ CD21^-^ | 0.0 (0.0-0.0) | 0.0. (0.0-0.1) | 0.0 (0.0-0.2) |
|  | IgM^+^ IgD^+^ memory CD27^+^ CD21^+^ | 0.0 (0.0-0.5) | 0.0 (0.0-1.2) | 0.2 (0.0-1.3) |
|  | IgM^+^ IgD^-^ memory CD27^-^ CD21^+^ | 0.0 (0.0-0.0) | 0.0 (0.0-0.1) | 0.0 (0.0-0.1) |
|  | IgM^+^ IgD^-^ memory CD27^+^ CD21^-^ | 0.0 (0.0-0.0) | 0.0 (0.0-0.0) | 0.0 (0.0-0.0) |
|  | IgM^+^ IgD^-^ memory CD27^+^ CD21^+^ | 0.0 (0.0-0.1) | 0.0 (0.0-0.3) | 0.1 (0.0-0.7) |
|  | IgG^+^ memory CD27^-^ CD21^-^ | 0.0 (0.0-0.0) | 0.0 (0.0-0.2) | 0.0 (0.0-0.2) |
|  | IgG^+^ memory CD27^-^ CD21^+^ | 0.0 (0.0-0.0) | 0.0 (0.0-0.2) | 0.0 (0.0-0.2) |
|  | IgG^+^ memory CD27^+^ CD21^-^ | 0.0 (0.0-0.0) | 0.0 (0.0-0.2) | 0.0 (0.0-0.1) |
|  | IgG^+^ memory CD27^+^ CD21^+^ | 0.0 (0.0-0.2) | 0.0 (0.0-0.8) | 0.2 (0.0-1.0) |
|  | IgA^+^ memory CD27^-^ CD21^-^ | 0.0 (0.0-0.1) | 0.0 (0.0-0.1) | 0.0 (0.0-0.1) |
|  | IgA^+^ memory CD27^-^ CD21^+^ | 0.0 (0.0-0.0) | 0.0 (0.0-0.0) | 0.0 (0.0-0.1) |
|  | IgA^+^ memory CD27^+^ CD21^+^ | 0.0 (0.0-0.2) | 0.0 (0.0-0.4) | 0.1 (0.0-0.5) |
|  | IgG^+^ circulating PC | 0.0 (0.0-0.0) | 0.0 (0.0-0.8) | 0.0 (0.0-0.2) |
|  | IgA^+^ circulating PC | 0.0 (0.0-0.0) | 0.0 (0.0-0.5) | 0.0 (0.0-0.1) |

APC, antigen-presenting cells; CM, central memory; EM, effector memory; TEMRA, effector memory T-cell re-expressing CD45RA; Tfh, follicular helper T cell, Treg, regulatory T cell; Ig, immunoglobulin; PC, plasma cells.

Supplemental Table 5. Baseline distribution of the 10 main compartments, grouping all 56 immune cell types, used in the logistic regression model. Numbers indicate the median percentage, along with range, in the peripheral blood of patients with multiple myeloma (MM, n=28), patients with a B-cell lymphoproliferative disorder (B-CLPD, n=53), and health care practitioners (HCP, n=96).

| **Main immune cell compartment** | **MM** | **B-CLPD** | **HCP** |
| --- | --- | --- | --- |
| Basophils | 0.8 (0.0-4.2) | 0.8 (0.1-2.6) | 0.8 (0.0-2.4) |
| Eosinophils | 1.5 (0.2-10.3) | 1.8 (0.0-8.4) | 1.0 (0.0-4.0) |
| Neutrophils | 47.6 (20.8-73.7) | 54.9 (3.9-76.0) | 55.0 (32.8-83.0) |
| Classical monocytes | 11.7 (5-41.4) | 6.9 (0.3-21.0) | 5.7 (0.0-10.6) |
| Intermediate monocytes | 0.4 (0.0-4.5) | 0.4 (0.0-5.7) | 0.2 (0.0-11.9) |
| Non-classical monocytes | 0.3 (0.0-1.6) | 0.6 (0.0-2.3) | 0.5 (0.0-1.9) |
| Dendritic cells | 0.4 (0.1-1.4) | 0.4 (0.0-2.7) | 0.5 (0.0-1.0) |
| CD4^+^ T cells | 8.2 (1.0-18.1) | 10.9 (1.3-51.6) | 16.4 (4.5-26.6) |
| CD8^+^ T cells | 14.3 (1.8-36.4) | 6.5 (0.8-21.6) | 6.4 (1.8-17.4) |
| B cells | 0.3 (0.0-5.7) | 1.4 (0.0-8.5) | 3.2 (0.0-18.9) |

Supplemental Table 6. Optimal cutoffs in percentages and absolute counts (x10^3^ cells/µL) for each group of immune cell types included in the predictive model.

|  | **Percentages in peripheral blood** | | | **Absolute counts (x10^3^ cells/µL)** | | |
| --- | --- | --- | --- | --- | --- | --- |
| **Main immune cell type** | **Cut-off** | **AUC** | ***P*-value** | **Cut-off** | **AUC** | ***P*-value** |
| Basophils | 0.24 | 0.477 | 0.665 | 42 | 0.567 | 0.231 |
| Eosinophils | 0.01 | 0.394 | **0.047** | 22 | 0.451 | 0.378 |
| Neutrophils | 48.44 | 0.447 | 0.323 | 2783 | 0.631 | **0.018** |
| Classical monocytes | 0.12 | 0.552 | 0.057 | 21 | 0.586 | 0.124 |
| Intermediate monocytes | 0.08 | 0.381 | **0.026** | 7 | 0.461 | 0.482 |
| Non-classical monocytes | 2.67 | 0.331 | **0.002** | 265 | 0.442 | 0.298 |
| Dendritic cells | 0.40 | 0.699 | **< 0.001** | 21 | 0.769 | **< 0.001** |
| CD4^+^ T cells | 12.43 | 0.743 | **< 0.001** | 915 | 0.778 | **< 0.001** |
| CD8^+^ T cells | 4.16 | 0.477 | 0.661 | 223 | 0.543 | 0.440 |
| B cells | 1.78 | 0.800 | **0.047** | 128 | 0.831 | **<0.001** |

Supplemental Table 7. Description of the antibodies and reagents used in this study.

| **Panel** | **Antigen** | **Label** | **Clone** | **Cat #** | **Company** |
| --- | --- | --- | --- | --- | --- |
| Granulocytes and antigen-presenting cells | CD36 | FITC | CLB-IVC7 | IM1613 | Sanquin |
|  | SLAN | PE | DD-1 | 130-093-029 | MB |
|  | CD33 | PerCP-Cy5.5 | P67.6 | 333146 | BDB |
|  | CD16 | PE-Cy7 | 3G8 | 560716 | BDB |
|  | CD123 | APC | AC145 | 130-090-901 | MB |
|  | CD14 | APCH7 | MϕP9 | 641394 | BDB |
|  | HLA-DR | Pacific Blue | L243 | 307633 | Biolegend |
|  | CD45 | OC515 | GA90 | CYT-450C | Cytognos |
| T-cells | CD45RA | FITC |  | 335039 | BDB |
|  | CD25 | PE | 2A3 | 341011 | BDB |
|  | CD8 | PerCP-Cy5.5 | SK1 | 341050 | BDB |
|  | CD279 (PD1) | PE-Cy7 | PD1.3 | A78885 | BC |
|  | CXCR5 | APC |  | FAB190A | R&D |
|  | CD197 (CCR7) | APC-Cy7 |  | 353212 | Biolegend |
|  | CD4 | Pacific Blue | 13B8.2 | B49197 | BC |
|  | CD127 | BV510 | HIL-7R-M21 | 563086 | BDB |
| B-cells | IgE+IgA | FITC |  | H15701 / 130-093-071 | TF / MB |
|  | IgG+IgA | PE | G18-145 | 555787 / 130-093-128 | BDB / MB |
|  | IgD | PerCP-Cy5.5 | IA6-2 | 348208 | Biolegend |
|  | CD19 | PE-Cy7 | J3-119 | IM3628 | BC |
|  | CD21 | APC | B-ly4 | 559867 | BDB |
|  | CD38 | APCH7 | HB7 | 656646 | BDB |
|  | CD27 | BV421 | M-T271 | 562513 | BDB |
|  | IgM | BV510 | MHM-88 | 314521 | Biolegend |
| HLA typing | HLA-A24 | PE | 22E1 | LS-C179737 | LSBio |
|  | HLA-B7 | PE | BB7.1 | 566654 | BDB |
|  | HLA-A3 | PE | GAP.A3 | 12-5754-42 | TF |
|  | HLA-A2 | BV421 | BB7.2 | 740082 | BDB |
| Virus-specific CD8^+^ T-cells | CD3 | BUV395 | SK7 | 564001 | BDB |
|  | CD4 | BUV496 | SK3 | 612936 | BDB |
|  | CD45RA | BUV563 | HI100 | 612926 | BDB |
|  | CD8 | BUV805 | SK1 | 612889 | BDB |
|  | CD45 | Pacific Orange | HI30 | MHCD4530 | TF |
|  | CCR7 | BV605 | 2-L1-A | 566754 | BDB |
|  | PD1 | BV650 | EH12.1 | 564104 | BDB |
|  | Dextramer cocktail | PE |  | RX08 | Immudex |
|  | TIGIT | APC | A15153G | 372706 | Biolegend |

BDB: BD Biosciences (San Jose, CA); BC. Beckman Coulter (Indianapolis, IN); Biolegend (San Diego, CA); Cytognos (Salamanca, Spain); Immudex (Virum, Denmark); LSBio: LifeSpan Biosciences (Seattle, WA); MB: Miltenyi Biotec (Bergisch Gladbach, Germany); R&D systems (Minneapolis, MN); Sanquin (Amsterdam, Netherlands); TF: Thermo Fisher (Waltham, MA).

**Supplemental Figures**

Supplemental Figure 1**. Relative distribution of the 56 immune cell types systematically identified in all individuals at all time points.** Percentages of **(A)** granulocytic, **(B)** antigen-presenting-cell, **(C)** CD4^+^ T-cell, **(D)** CD8^+^ T-cell, and **(E)** B-cell subsets in peripheral blood before vaccination (baseline), 7 (1^st^ D+7) and 14 (1^st^ D+14) days after the first dose, and 7 (2^nd^ D+7) and 60 (2^nd^ D+60) days after the second dose of the SARS-CoV-2 vaccine were determined in 96 health care practitioners (HCP), 53 patients with a mature B-cell lymphoproliferative disorder (B-CLPD) and 28 patients with multiple myeloma (MM).

Blue, green and red asterisks indicate significant differences between indicated time points in HCP, patients with a B-CLPD, and patients with MM, respectively. Black asterisks with the table represent significant differences between groups at each time point. *, *P* <.05; **, *P* <.01; ***, *P* <.001. CM, central memory; CPC: circulating plasma cells; EM, effector memory; TEMRA, effector memory re-expressing CD45RA; Tfh, helper follicular T-cells; Treg; regulatory T-cells.

1. Granulocytes


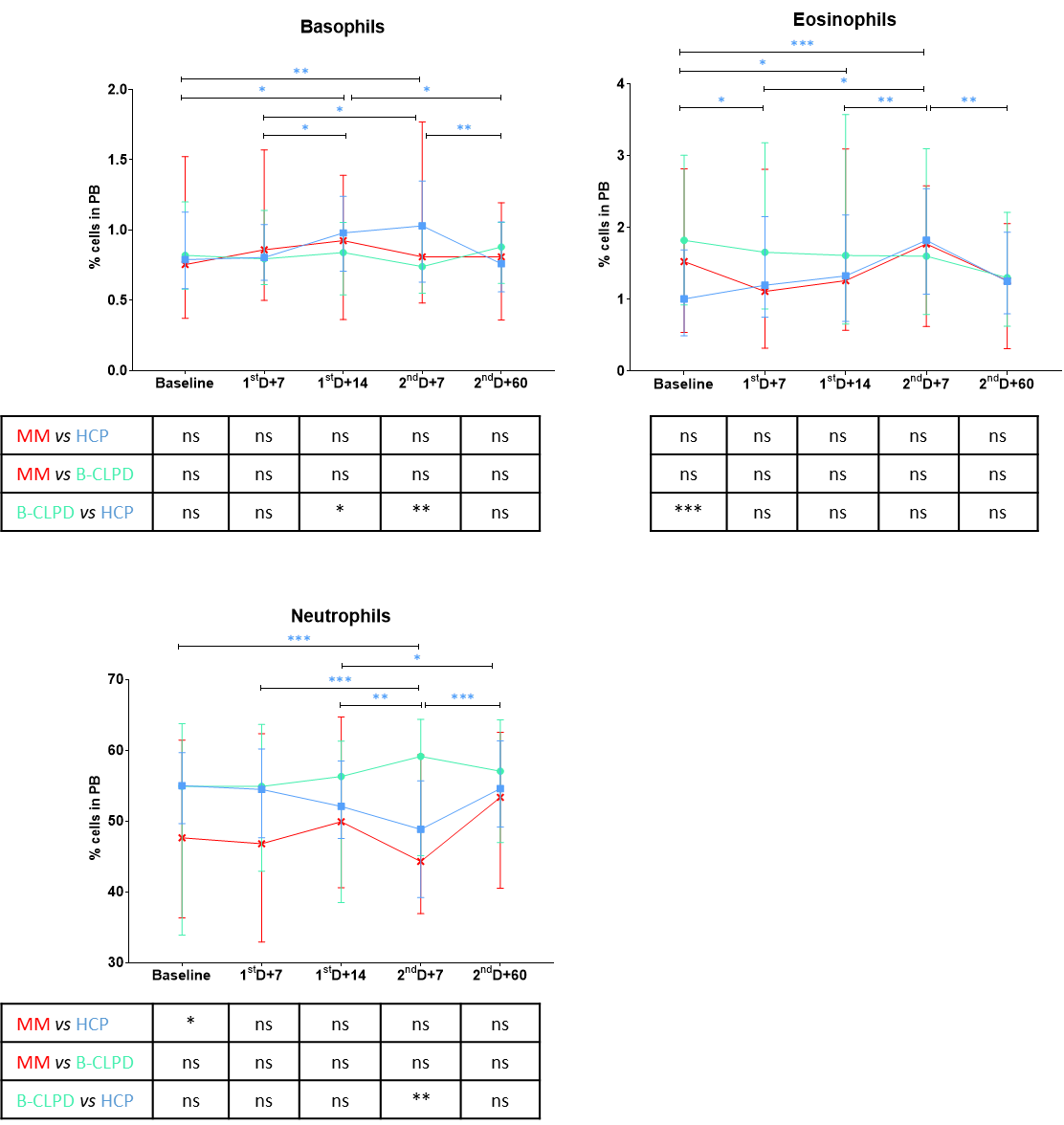


1. Antigen-presenting cells


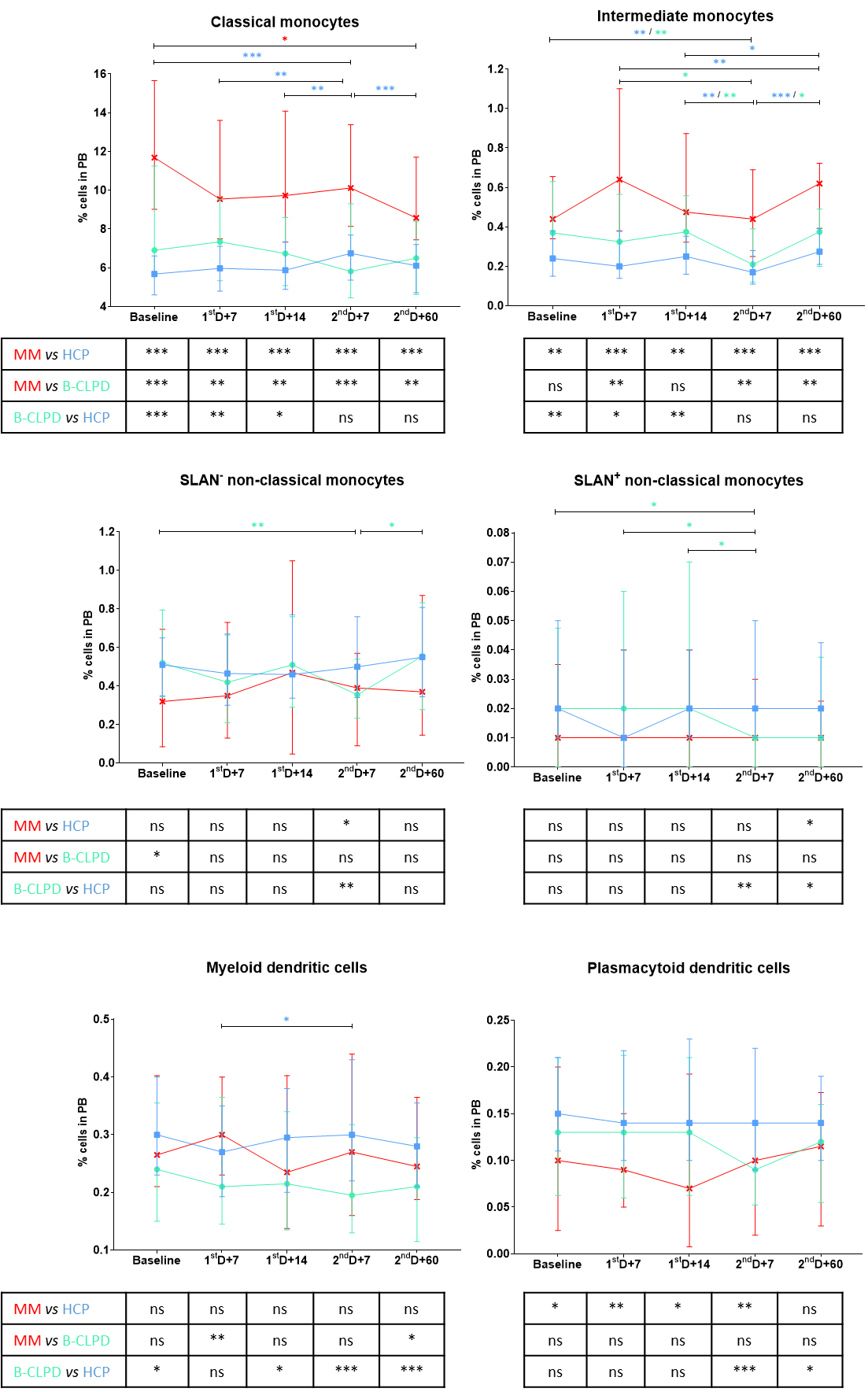


1. CD4^+^ T-cells


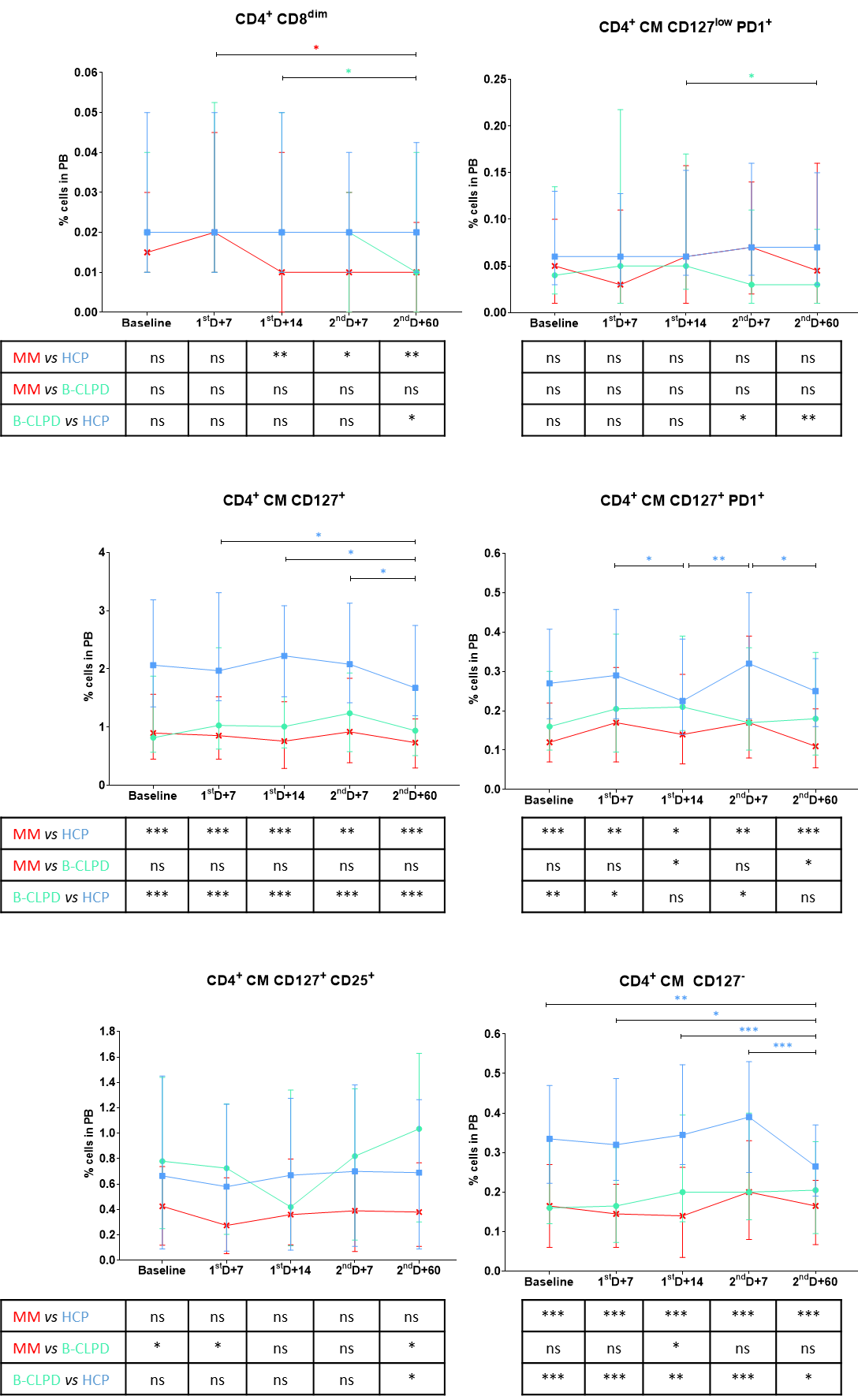


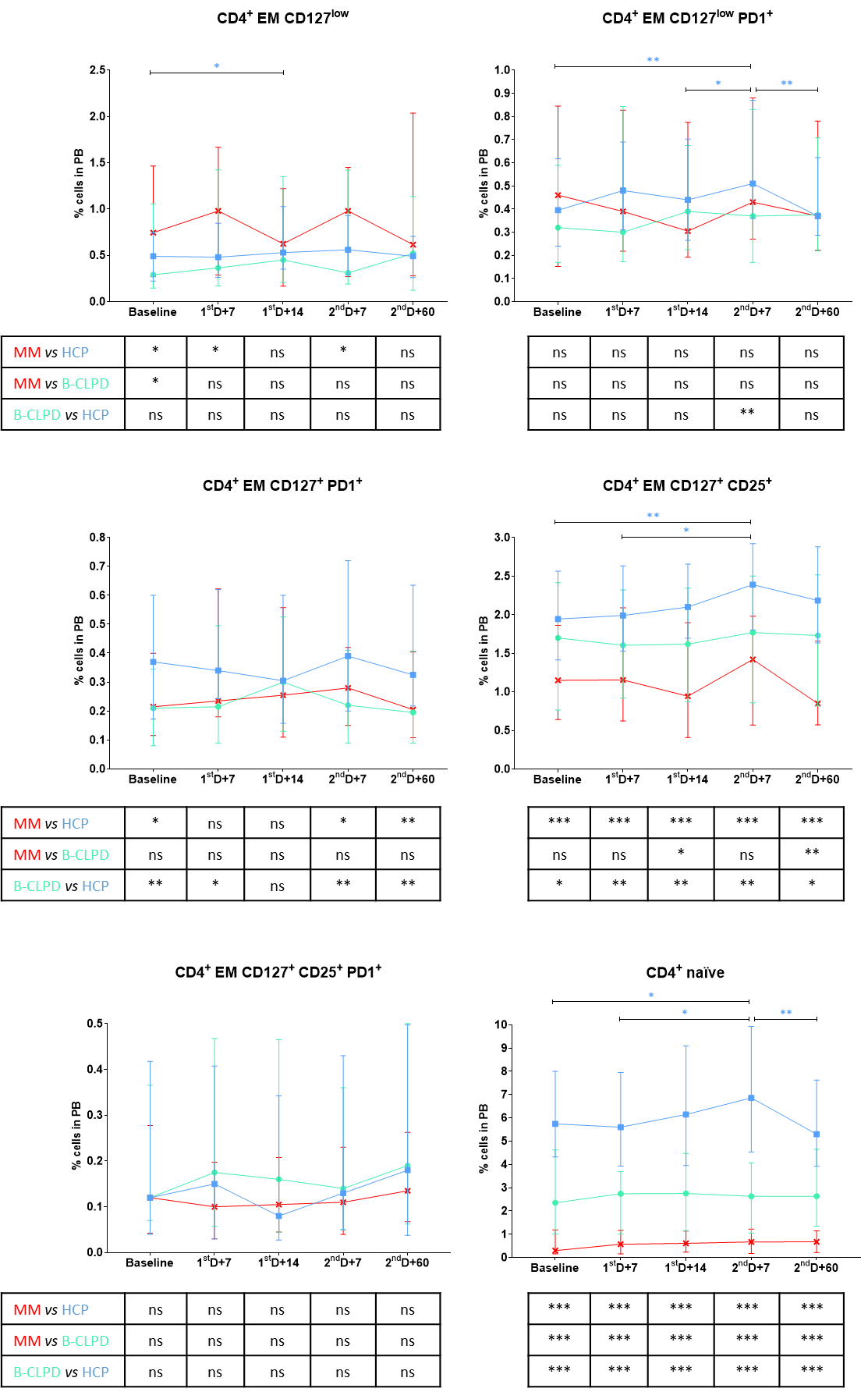


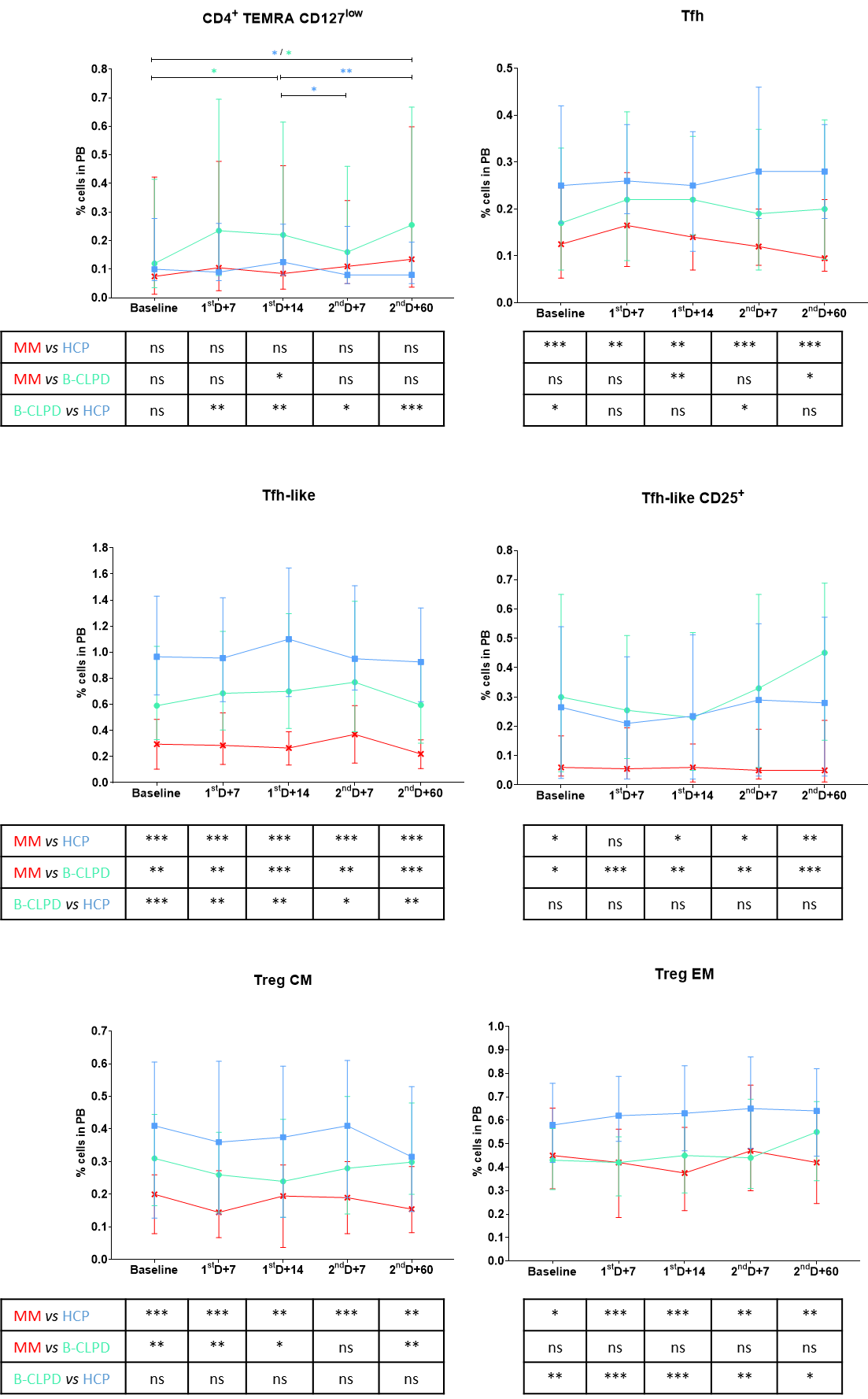


1. CD8^+^ T-cells


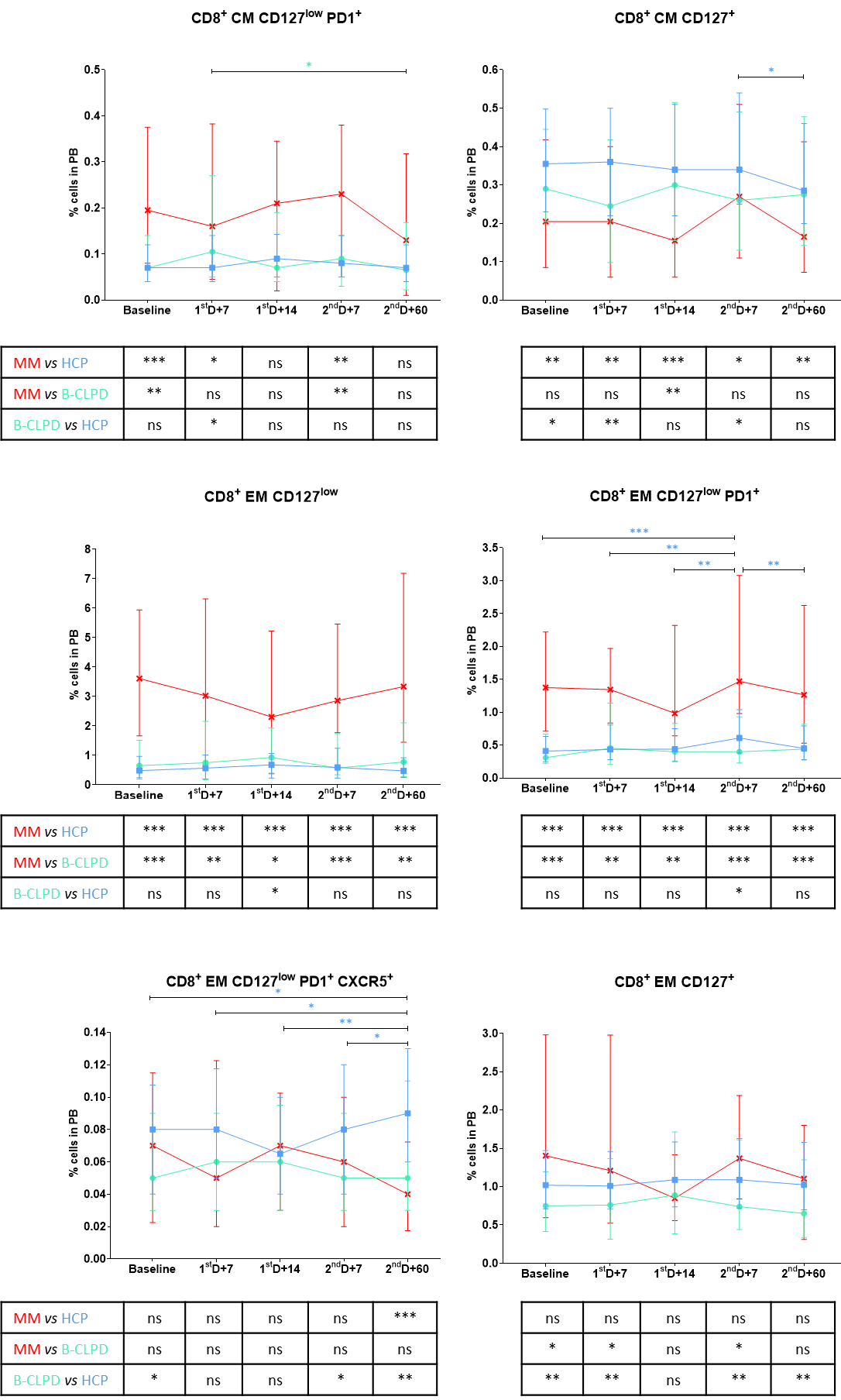


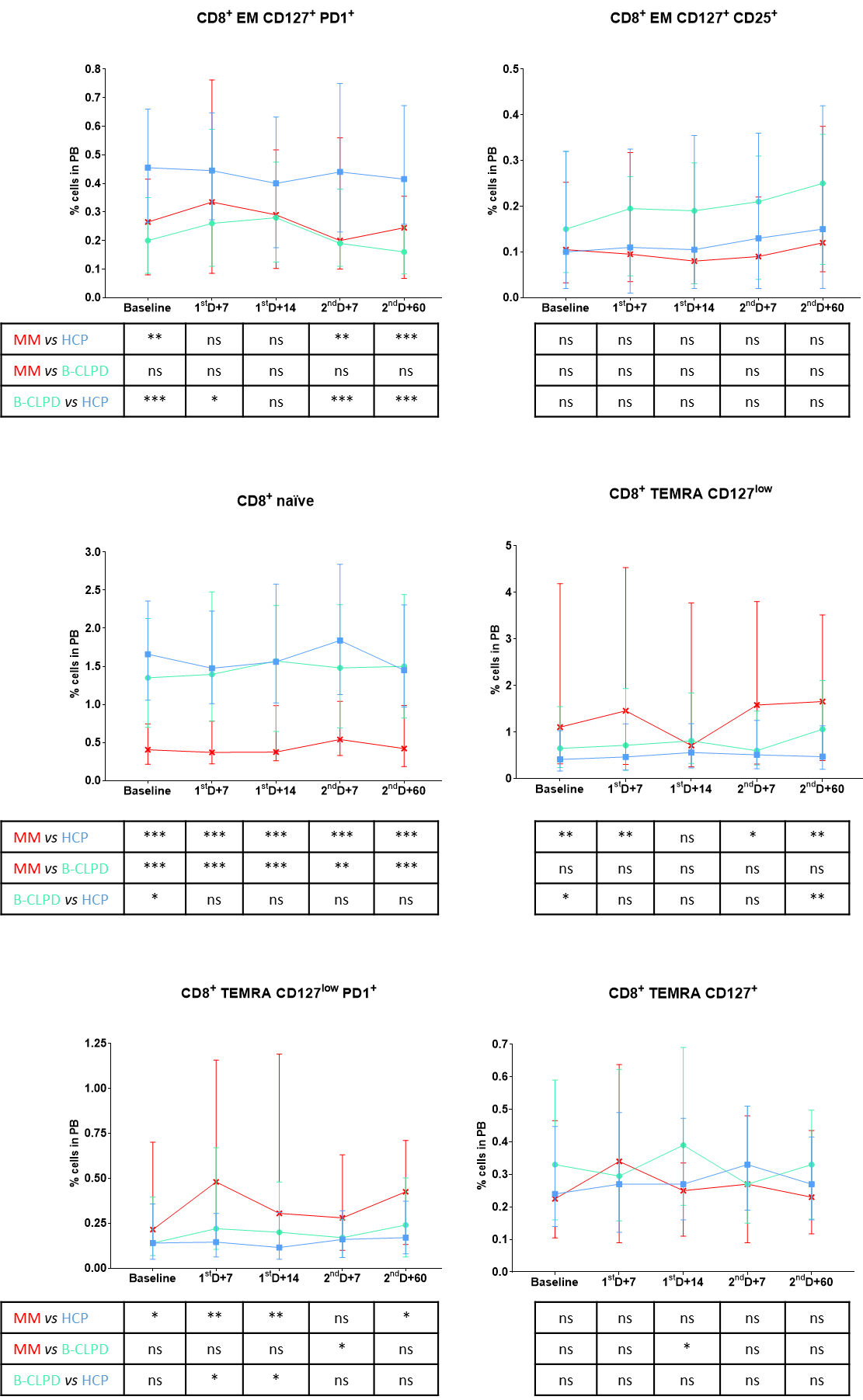


1. B-cells


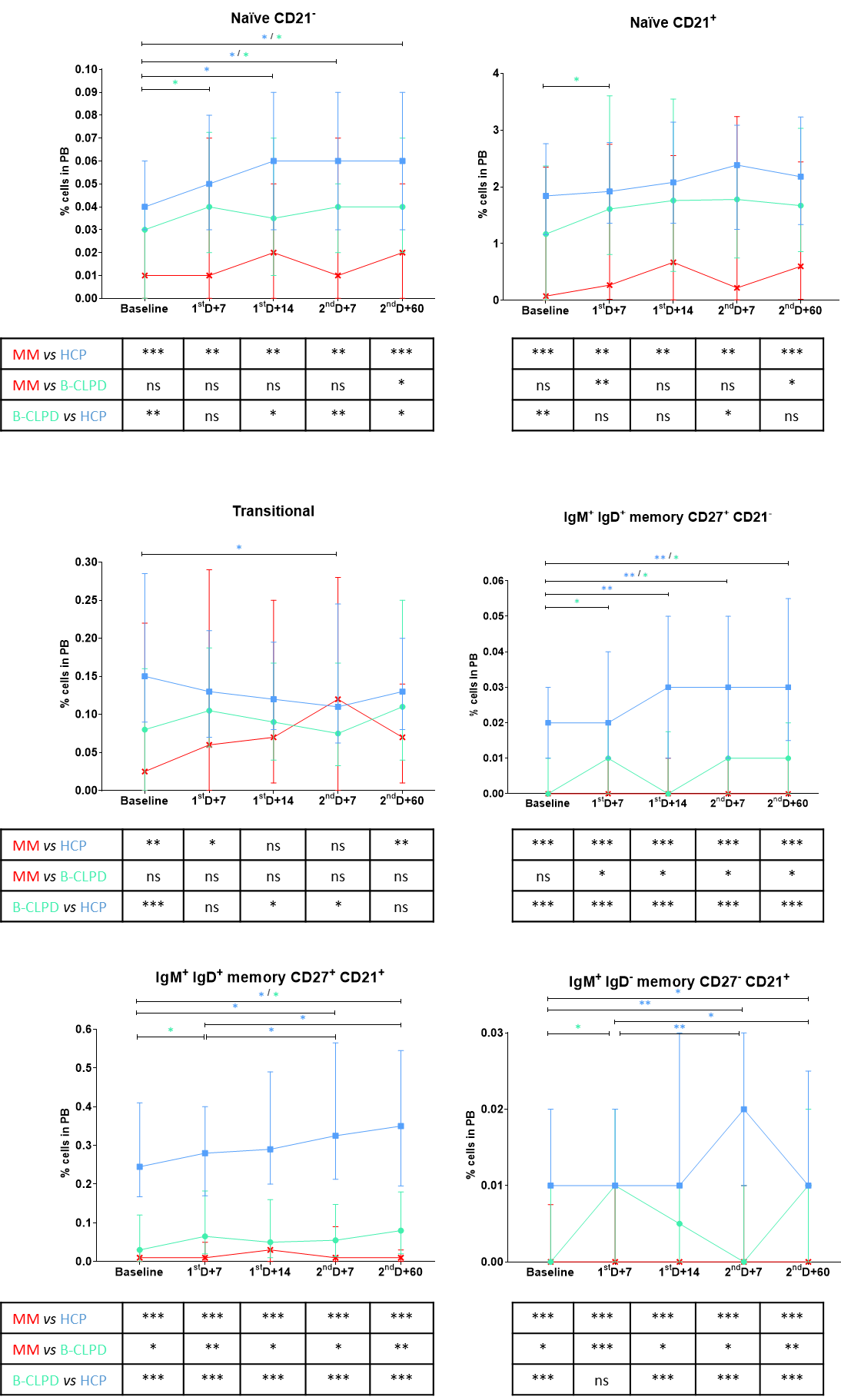


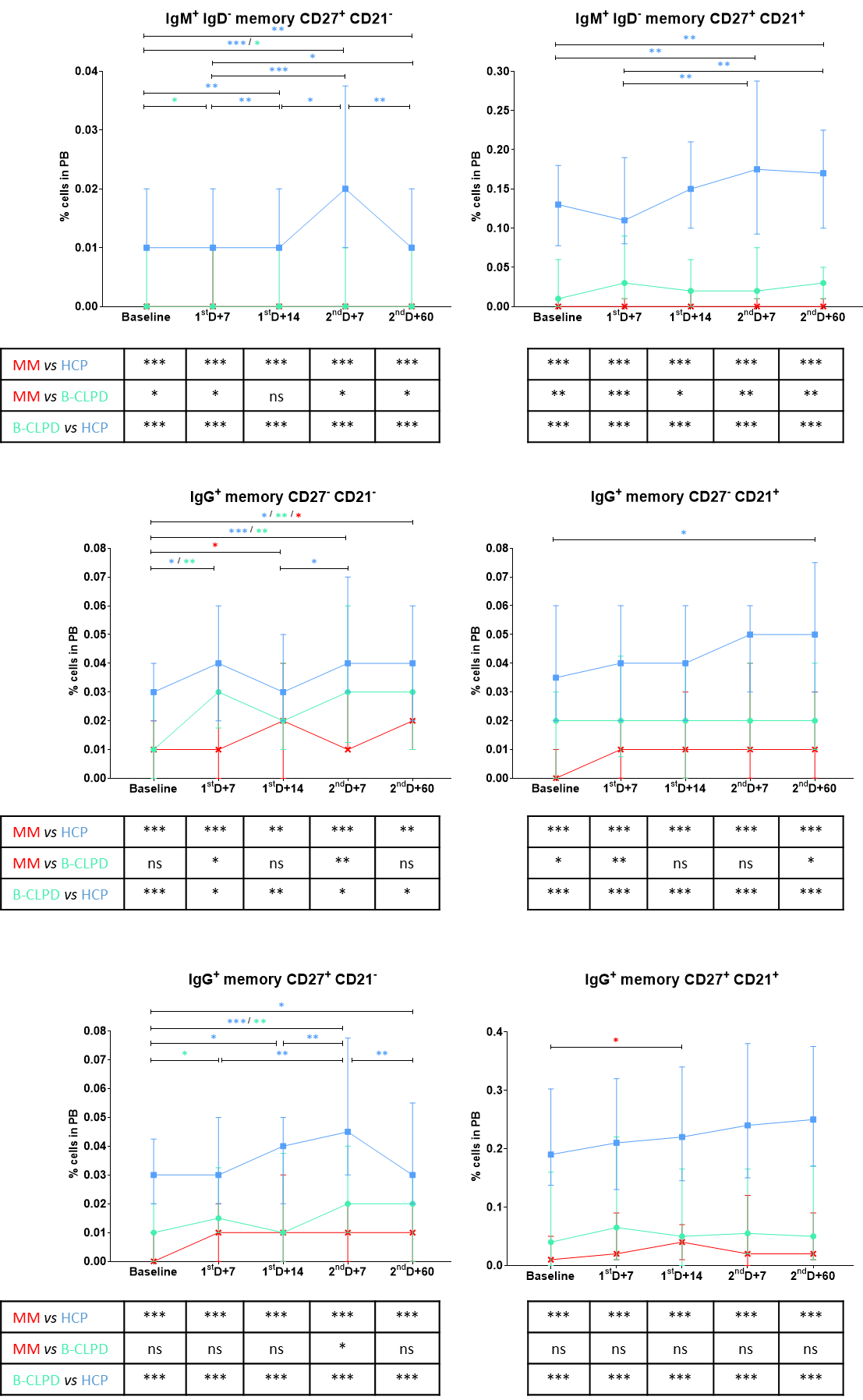


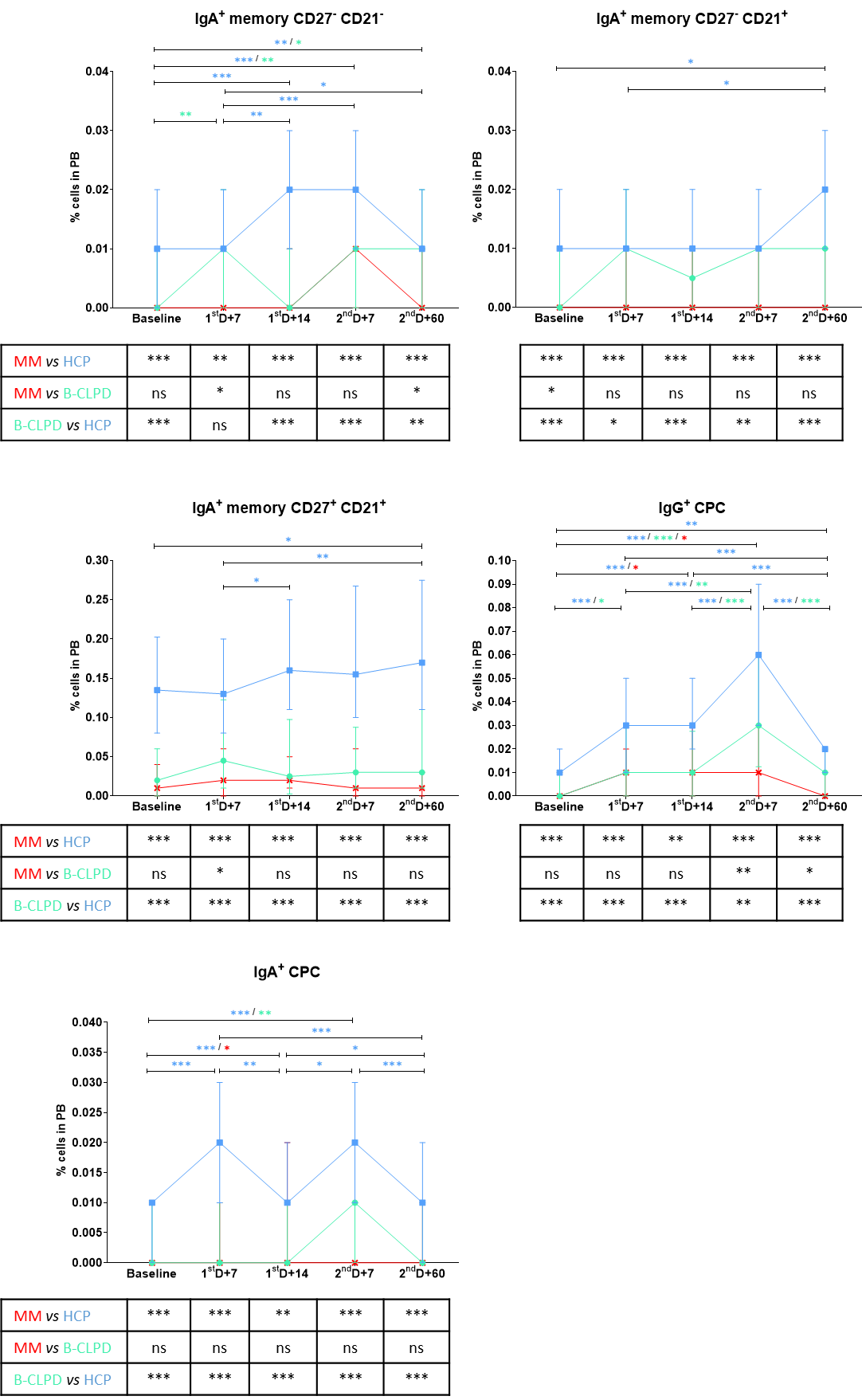


Supplemental Figure 2. **Longitudinal production of anti-spike (S) antibodies in response to COVID-19 vaccine.** Indexes of **(A)** IgM, **(B)** IgA, and **(C)** IgG antibodies, as well as **(D)** concentration of IgG against the stable trimer of the S-glycoprotein were calculated at baseline, 7 days after the first dose (1^st^ D+7), as well as 7 (2^nd^ D+7) and 60 (2^nd^ D+60) days after the second dose in health care practitioners (HCP; n=96), patients with a mature B-cell lymphoproliferative disorder (B-CLPD; n=53) and patients with multiple myeloma (MM; n=28). Vertical lines and syringes represent the two-dose administration of the primary series of vaccination. Blue, green and red asterisks represent significant differences between indicated time points in HCP, and B-CLPD and MM patients, respectively. Black asterisks within tables show significant differences in indicated comparisons at each time point.

*, *P* <.05; **, *P* <.01; ***, *P* <.001; ns, non-significant.


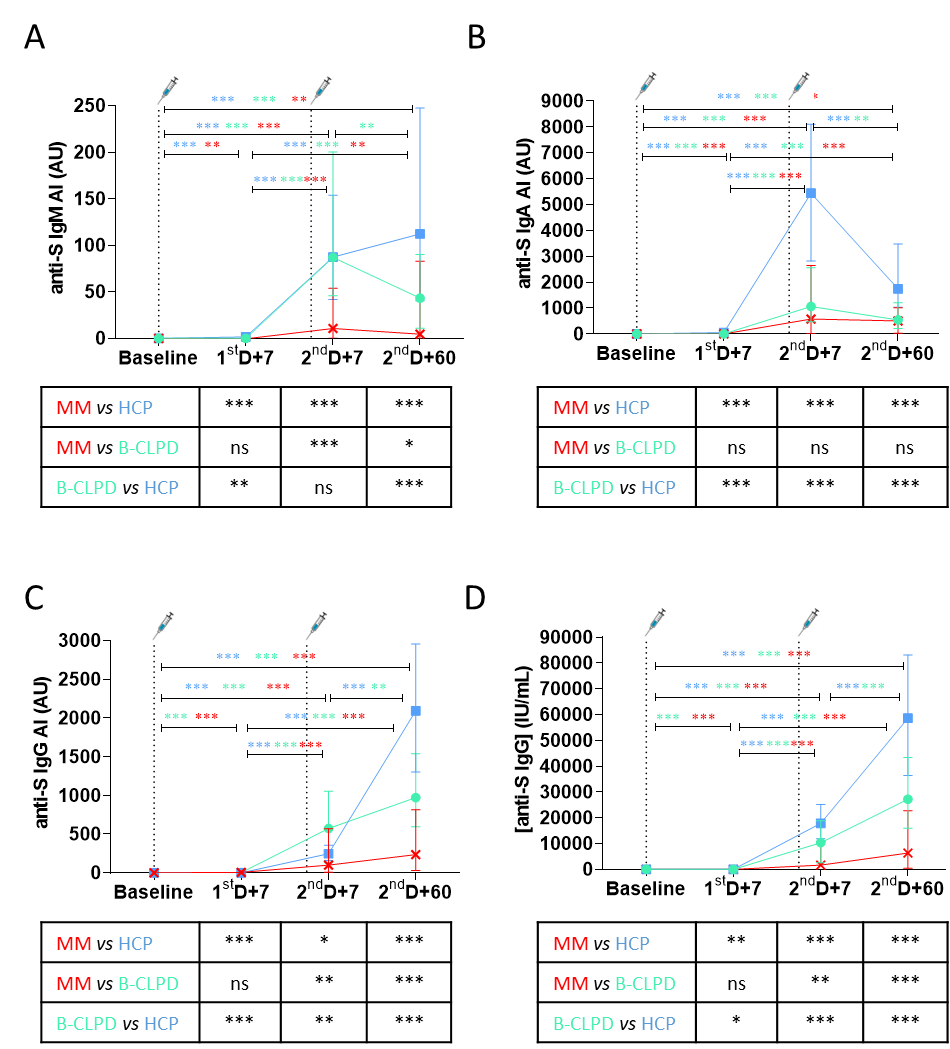


Supplemental Figure 3. **Cellular response to the COVID-19 vaccine booster dose.** **(A)** SARS-CoV-2-specific CD8^+^ T-cells were identified in HLA-A2 allele-carrying individuals 14 days after the booster (n=33 HCP; n=7 B-CLPD patients; n=7 MM patients). **(B)** Antigen-dependent differentiation of SARS-CoV-2-specific CD8^+^ T-cells was characterized before and after the booster using anti-CCR7 and anti-CD45RA antibodies to distinguish naïve (CCR7^+^ CD45RA^+^), central memory (CM, CCR7^+^ CD45RA^-^), effector memory (EM, CCR7^-^ CD45RA^-^), and effector memory T-cells re-expressing CD45RA (TEMRA, CCR7^-^ CD45RA^+^) cells. Asterisks indicate significant differences in each T-cell compartment between time points.


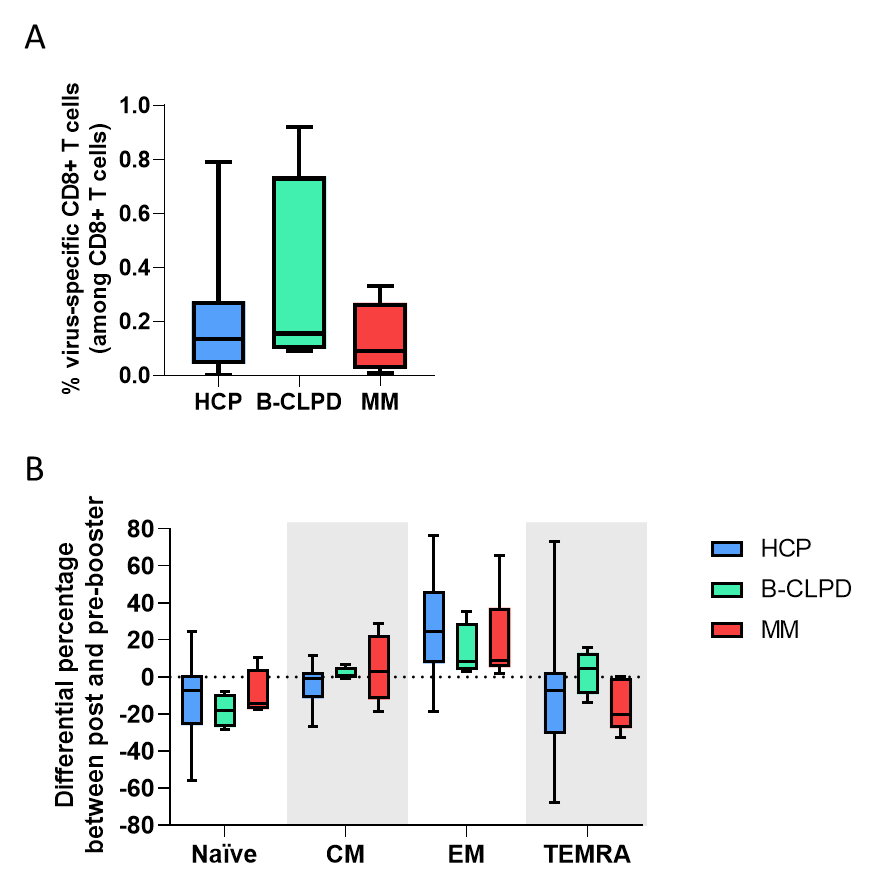


Supplemental Figure 4. **Predictive model of vaccine immunogenicity using absolute counts. (A)** Odds ratio multivariate analysis with 95% confidence intervals (CI) included in the logistic regression model. After a 10-fold cross-validation, the absolute counts of B cells and dendritic cells measured before vaccination significantly predicted inadequate seroconversion one week after the second dose, defined as below the median observed in patients at this time point. *, *P* <.05; **, *P* <.01. **(B)** Area under the curve (AUC) and significance of the prediction probabilities of the model based on absolute counts.


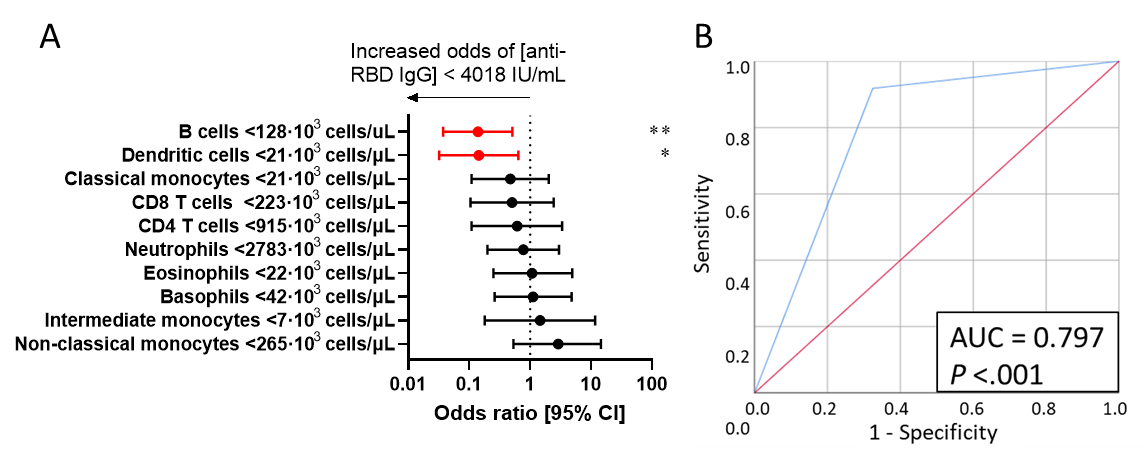


Supplemental Figure 5. **Humoral response to the COVID-19 vaccine booster dose.** Concentration of anti-RBD IgG was measured prior to and 14 days after the booster dose administration in HCP (n=91) and B-CLPD (n=33) and MM (n=17) patients. Blue asterisks within the graph indicate significant differences between time points in HCP; and black asterisks within tables show significant differences between groups of individuals at each time point.

***, *P* <.001; ns, non-significant


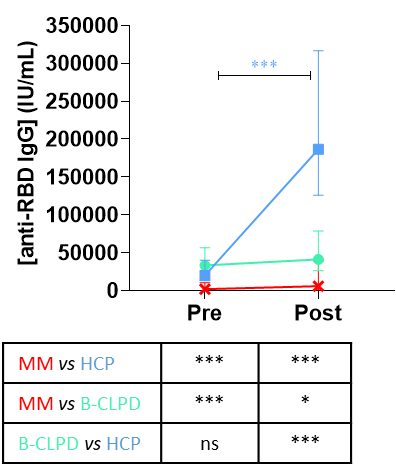


Supplemental Figure 6. **Gating strategy of the 56 immune cell types systematically identified in all samples by automated clustering.** Immune cell types were identified based on their size (FSC), complexity (SSC), and the expression of several markers: **(A)** CD45, HLA-DR, CD123, CD33 and CD16 for main immune cell types: granulocytes (i.e., eosinophils, neutrophils, and basophils), antigen presenting cells (APC) and lymphocytes; **(B)** CD14, CD16, CD45, CD36, CD123, HLA-DR and SLAN for APC, which were further classified into: classical monocytes, intermediate monocytes, SLAN^-^ and SLAN^+^ non-classical monocytes, and myeloid and plasmacytoid dendritic cells (mDC and pDC, respectively); **(C)** CD27, CD38, CD21 and immunoglobulins D, M, A and G (IgD, IgM, IgA and IgG, respectively) were used for B-cell categorization within the CD19^+^ compartment; and **(D)** CD4, CD8, CD127, CD25, CCR7, CD45RA, PD1 and CXCR5 for T-cell subsets within both CD4 and CD8 compartments.

CM, central memory; CPC: circulating plasma cells; EM, effector memory; TEMRA, effector memory re-expressing CD45RA; Tfh, helper follicular T-cells; Treg; regulatory T-cells.


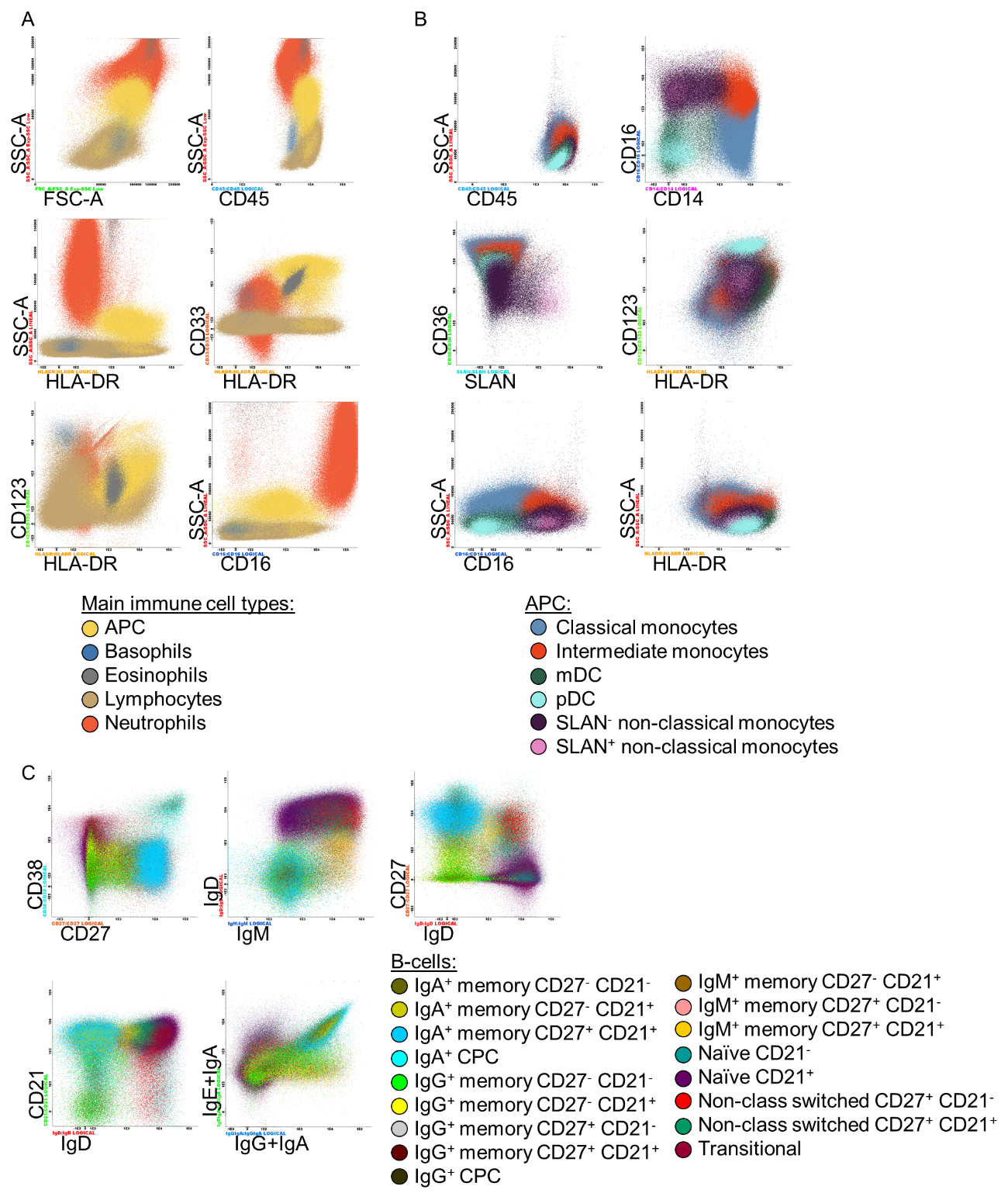


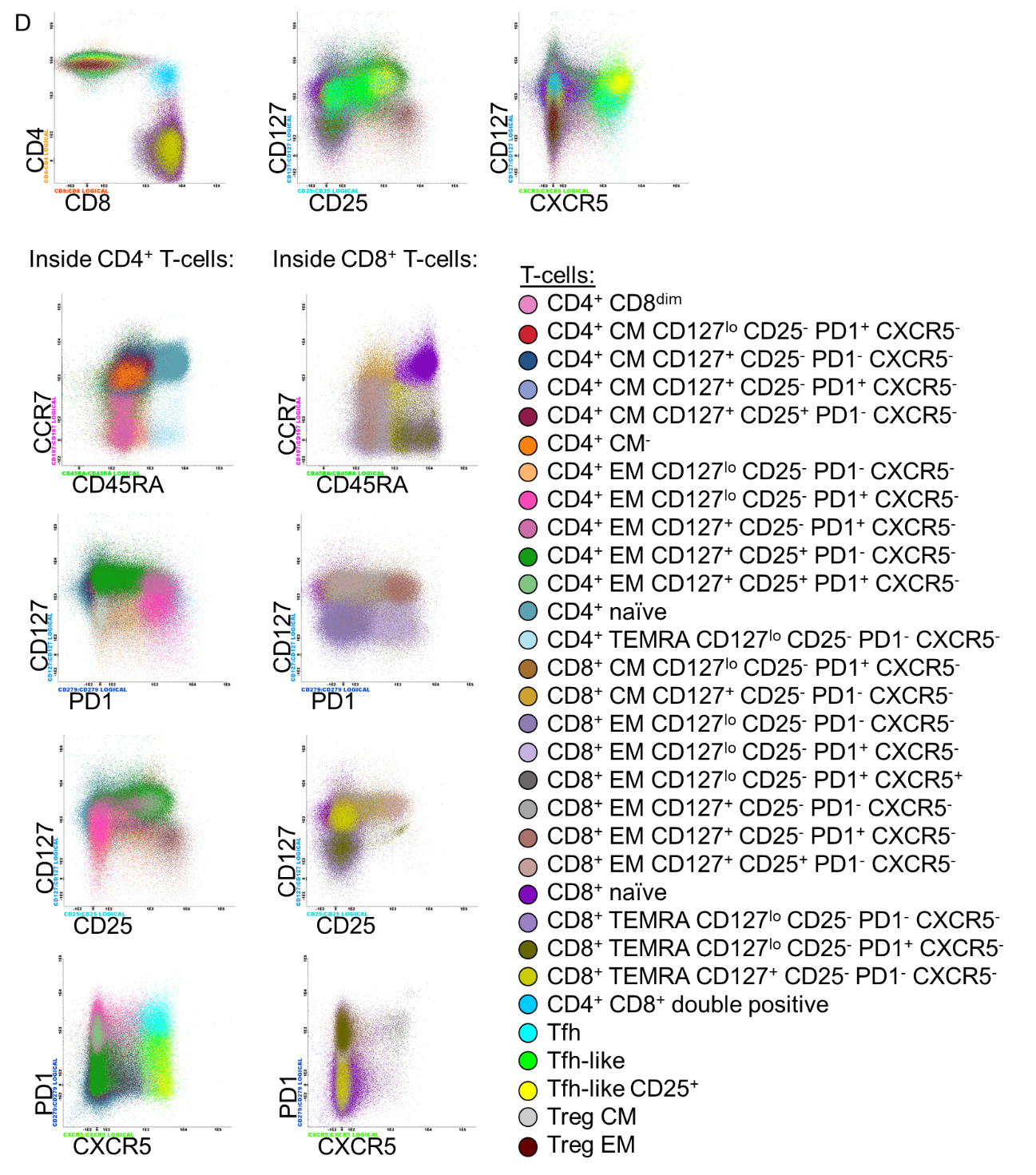

Supplement: Supplementary file 1 — Supplemental Material [file 41408_2024_1089_MOESM1_ESM.docx]
